# Origins of Susceptibility to Insect Herbivores in High-Yielding Hybrid and Inbred Rice Genotypes

**DOI:** 10.3390/insects15080608

**Published:** 2024-08-12

**Authors:** Finbarr G. Horgan, Maria Liberty P. Almazan, Carmencita C. Bernal, Christine Jade Dilla-Ermita, Goli Ardestani, Enrique A. Mundaca, Eduardo Crisol-Martínez

**Affiliations:** 1 EcoLaVerna Integral Restoration Ecology, Bridestown, Kildinan, T56 P499 County Cork, Ireland; ecrisol@coexphal.es; 2 School of Agronomy, Faculty of Agrarian and Forest Sciences, Catholic University of Maule Casilla 7-D, Curicó 3349001, Chile; emundaca@gmail.com; 3 Centre for Pesticide Suicide Prevention, University/BHF Centre for Cardiovascular Science, University of Edinburgh, Edinburgh EH16 4TJ, UK; 4 International Rice Research Institute, Makati 1226, Manila, Philippines; 5 Department of Plant Sciences, UC Davis, One Shields Ave., Davis, CA 95616, USA; 6 Boston IVF—IVIRMA Global Research Alliance, Waltham, MA 02451, USA; 7 Association of Fruit and Vegetable Growers of Almeria (COEXPHAL), Carretera de Ronda 11, 04004 Almeria, Spain

**Keywords:** brown planthopper, fertilizer, host plant resistance, rice breeding, rice herbivores, rice phylogeny, whitebacked planthopper, yellow stemborer

## Abstract

**Simple Summary:**

Hybrid rice is grown by millions of Asian farmers and has normally higher yields compared to inbred varieties. However, hybrid rice has been associated with high damage from herbivores. This susceptibility could be due to the physiology of the hybrid plant type or due to a limited availability of male sterile parents that are necessary for hybrid seed production. We examined how plant type and breeding affect the relative susceptibilities of hybrid and inbred rice by exposing 32 rice genotypes to the brown planthopper, whitebacked planthopper, and yellow stemborer in controlled environments and field plots. We compared insect fitness on the plants and related this to the type and genetic similarity of genotypes. Despite their diverse origins (China, Colombia, India, and the Philippines), the hybrids and inbreds formed two distinct genetic groups, thereby confounding plant type and phylogeny. Hybrids were generally more susceptible to stemborers but not to planthoppers. Relative herbivore fitness was strongly influenced by plant origin (i.e., breeding program) with one group of related genotypes being relatively susceptible to all three herbivores. Our results indicate that hybrids are not inherently more susceptible than inbreds to insect herbivores and that careful screening with the elimination of the most susceptible genotypes is necessary to reduce herbivore damage to hybrid and inbred rice in Asia.

**Abstract:**

Several studies have reported higher damage from insect herbivores to hybrid compared to inbred (pure line) rice. We used a collection of 20 hybrid and 12 inbred genotypes from diverse origins to test the hypotheses that hybrid rice susceptibility is due to (a) the hybrid plant type and/or (b) rice phylogeny. We challenged the genotypes with *Nilaparvata lugans* (BPH), *Sogatella furcifera* (WBPH) and *Scirpophaga incertulas* (YSB) in greenhouse and screenhouse bioassays and monitored herbivores in field plots. We used single nucleotide polymorphic (SNP) markers to assess genetic similarities between the genotypes and found that the hybrids and inbreds formed two distinct clusters regardless of origin. In the screenhouse, hybrids were more susceptible than inbreds to YSB; however, resistant hybrids and susceptible inbreds were also apparent from both the screenhouse and field plots. Plant biomass was the best predictor of susceptibility to YSB. Plant origin had a greater effect than plant type on susceptibility to BPH and WBPH. WBPH was the most abundant planthopper in the field plots where numbers were highly correlated with planthopper fitness in the greenhouse bioassays. Our results provide evidence that high-yielding hybrids that are relatively resistant to herbivores can be achieved through careful breeding. The avoidance of susceptible genotypes during breeding should remain a key element of integrated rice pest management.

## 1. Introduction

Hybrid rice was first developed in China in the 1960s after the discovery of a wild abortive cytoplasmic male sterile (WA-CMS) rice line [1,2,3]. The first hybrid varieties were subsequently released in the 1970s and rapidly gained prominence to eventually exceed 50% of total Chinese rice production [4,5]. More recently, hybrid rice has been promoted in India, Bangladesh, Vietnam, Myanmar, and the Philippines through the establishment of national hybrid rice breeding programs and through national and international private sector involvement [5,6]. In many cases, these breeding programs continued to rely on male sterile lines (the female parents) derived from the original Chinese WA-CMS [5], although newer breeding systems have also emerged in recent years [7,8,9]. Hybrid rice varieties often have higher yields (up to 10%) than comparable inbred varieties [4,10,11,12]. Because the F1 seeds segregate for sterility, hybrid seed production is restricted to breeding programs with access to male sterile lines and their associated maintainers (see below). Restricted seed production improves seed quality and avoids seed-borne diseases and weed contamination in farmers’ fields [5,13].

Despite the widescale promotion and rapid adoption of hybrid rice varieties in Asia, concerns have been raised about their relative susceptibility to pests when compared to inbred (also known as pure-line) varieties [5,13]. These issues have been linked to a high susceptibility or ‘hyper-susceptibility’ of the female parents (through the CMS lines) to planthoppers and leafhoppers and a high suitability of hybrids for stemborers due to hybrid physiology [5,11,14,15,16,17]. Outbreaks of the whitebacked planthopper, *Sogatella furcifera* (Horváth) have been associated with increasing areas of adoption of hybrid rice, particularly in China [16,17]. Similarly in tropical regions, the brown planthopper, *Nilaparvata lugens* (Stål), and yellow stemborer, *Scirpophaga incertulas* (Walker), attain relatively high densities in fields of hybrid rice compared to inbred varieties [5,18]. Through careful breeding, including the selection of relatively resistant male parents (known as restorer lines—see below), hybrid resistance to pests has improved [11,19]. Furthermore, several breeding programs have introgressed planthopper and gall midge resistance genes into hybrid breeding lines using marker-assisted selection [20,21,22,23,24]. However, planthoppers and other insect herbivores are noted for their ability to adapt to major resistance genes and, despite progress, susceptible hybrid varieties without planthopper resistance are still widely available [25]. Furthermore, because hybrid rice often receives higher fertilizer and pesticide inputs compared to inbred varieties [18,26,27,28,29,30], pest outbreaks continue to be associated with some commercial hybrids [18,28]. Because of such differences in hybrid and inbred rice crop management [5], it is still unclear whether hybrids are generally more susceptible to herbivore pests than inbreds and to what level hybrid physiology or breeding origins (i.e., the specific male and female parents used) determine susceptibility.

In the present study, we use a collection of comparative (i.e., similar crop duration, moderate to high tillering, suitable for tropical climates), high-yielding hybrid and inbred rice varieties to test the hypotheses that hybrid rice is generally more susceptible to insect herbivores than inbred varieties, due to (a) the hybrid plant type (i.e., anatomical and physiological traits determined by heterosis for development and growth) or (b) breeding origin (i.e., phylogeny and breeding program). We, therefore, challenged the varieties with three insect herbivores, the brown planthopper (BPH), whitebacked planthopper (WBPH), and yellow stemborer (YSB) in a series of greenhouse and screenhouse experiments and assessed their population densities in a related field plot study. We predicted that if the hybrid plant type determined susceptibility, then hybrids would be more susceptible to the herbivores than inbreds—without effects of genotype phylogeny; alternatively, if phylogeny has a greater effect on susceptibility, without plant-type effects, then breeding would play a greater role in determining susceptibility. To examine phylogenetic effects, we analyzed single nucleotide polymorphic (SNP) markers among the genotypes and used genotype origins in ordination analyses with plant type (hybrid/inbred) and comparative fitness traits from herbivore-antibiosis and antixenosis bioassays. To our knowledge, this is the largest comparative study of the relative susceptibilities of hybrid and inbred rice to insect herbivores and the first to control for phylogenetic differences across test plants. We discuss our results in the context of reducing yield losses from insect herbivores in tropical hybrid rice.

## 2. Materials and Methods

### 2.1. Plant Materials

We used 32 high-yielding varieties in our experiments. These included 20 hybrid varieties and 12 elite inbred varieties. The materials were obtained from breeding programs and commercial companies without access to parental lines. Further details of the varieties can be found in a paper by Bueno and Lafarge (2017) [10]. The identities of most of the varieties are not disclosed, under agreements with seed suppliers. The varieties were selected based on preliminary screening for yields and growth characteristics and included materials with diverse genetic backgrounds and from a range of geographical origins (Table S1). The selection of varieties was not based on any criteria related to pest or disease resistance/tolerance. During field plot studies at the International Rice Research Institute (IRRI), the hybrids yielded between 8 and 9.5 tons ha^−1^ in the dry season and 5 to 7.5 tons ha^−1^ in the wet season; the inbreds yielded between 7.5 and 9 tons ha^−1^ in the dry season and 5 to 8 tons ha^−1^ in the wet season [10].

Experiments were conducted in a greenhouse, screenhouse, and in field plots. Greenhouse experiments are useful for understanding planthopper-rice interactions because they avoid the normally high predation rates that occur under field conditions [31,32,33]. For stemborers, screenhouse studies were conducted because plants grown in the screenhouse are similar (tiller numbers, growth rates, development) to those grown in the field and stemborer-rice interactions are largely determined by plant anatomy [11,34]. Figure S1 presents a schematic of the different experiments and bioassays conducted during this study. We limited our experiments to early rice growth stages (i.e., before the booting stage) to avoid root competition in the greenhouse [35] and because planthoppers and stemborers mainly attack early rice crop stages. Furthermore, the general resistance of rice increases at older stages due to stem hardening and an increase in the C:N (carbon-nitrogen) ratios of tissues as plants mature [36,37,38,39].

We examined the early development of each variety in screenhouse plots. The pre-germinated seed was first sown to plastic basins (30 × 45 × 20 cm, W × L × H) and, after 10 days, was transplanted at one seedling per hill to a screenhouse facility (average temperature = 26 °C, relative humidity = 85%, 12 h day: 12 h night). The screenhouse had a series of concrete bays (2 × 20 m, W × L) that were open to the ground. The bays were filled with paddy soil (approx. 60 cm deep) from the field station. The whole facility was covered by 1 mm mesh. The plants were planted in microplots with one plant for each variety. Half of the plots were treated with 40 Kg N ha^−1^ applied basally with the remaining plots untreated. The plants were allowed to develop until 35 DAT, at which time a SPAD reading (chlorophyll content) was taken and the plants were destructively sampled by carefully pulling the plants from the soil and washing the roots under running water. The harvested plants were individually placed in paper bags and dried in a forced draught oven at 60 °C until a constant weight, after which time, the number of tillers was recorded, the plants were divided into roots and shoots, and the different plant parts were weighed using a precision balance.

### 2.2. Insect Herbivores

YSB mainly attacks seedlings or tillering rice and is the main stemborer species in tropical Asia. The adults deposit egg masses on the rice foliage. On hatching, the larvae tunnel into the rice tillers where they remain to feed and develop, eventually emerging from the tillers as adults [18,34]. YSB adults were collected 3–5 days before they were required for the experiments, from rice fields in Laguna Province using sweep-nets. The adults were placed in plastic cages (100 × 50 × 50 cm: H × W × L) with >30 DAS TN1. The adults were allowed to mate and lay eggs for up to 7 days. The egg masses were collected and placed in Eppendorf tubes until the larvae emerged. After hatching, the larvae were used in experiments within 1 h.

BPH and WBPH are regarded among the most damaging herbivores in lowland irrigated rice production systems throughout Asia [17,27,28]. An increasing prevalence of WBPH in recent decades has been associated with hybrid rice and was linked to the cytoplasm of the WA-CMS lines [16,17]. BPH has also been associated with hybrid rice production in previous studies [5] and both species are favored by relatively high fertilizer use and by the depletion of natural enemy numbers where pesticides are used excessively [29,30,40,41]. Both species attack early-stage rice plants (tillering stage) where they feed and lay eggs, but BPH can continue to oviposit and develop on rice until grain matures [42,43]. The nymphs of both species pass through five instars and under tropical conditions can have several generations per crop. Under favorable conditions, planthoppers produce larger numbers of brachypterous adults (males and females in BPH, females in WBPH). Macropterous adults emerge as the rice nears maturity or on low-quality hosts [43] and are capable of migrating 1000 s of Kilometers during the late spring and early summer from overwintering areas in tropical regions to temperate rice-growing regions [28,44]. Heavy infestations produce patches of dead plants in rice fields (known as hopperburn) [27].

The planthopper colonies were each initiated with >500 individuals collected from rice paddies in Laguna, Philippines. The founders were collected five years prior to the experiments using sweep-nets. The colonies were maintained in a shaded greenhouse in wire-mesh cages of 120 × 60 × 60 cm (H × W × L). The planthoppers were continuously fed with >30 DAS TN1, with plants changed every two weeks. Adults and nymphs used in the experiments were collected from the colonies using hand-held pooters. Details of virulence adaptations among Laguna BPH and WBPH were presented by Horgan et al. (2017) [45].

### 2.3. Genotyping

#### 2.3.1. Collection of Leaf Samples

Pre-germinated seeds of the 32 genotypes were individually sown in size-6 pots (15 × 15 cm, H × D) in a greenhouse at IRRI. The pots were filled with paddy soil that was mixed with basal amounts of ammonium phosphate fertilizer (0.8 g/pot) and watered each day. Seeds were covered with acetate cages (123 × 12 cm, H × D) to exclude herbivores without using pesticides. Temperatures in the greenhouse fluctuated between 26–40 °C and relative humidity between 76–89%, and plants were grown under natural light conditions (12 h day: 12 h night) [35]. Samples of young leaves were collected from plants at the 3 to 4-leaf stage and placed in 2 mL micro tubes with liquid nitrogen (−196 °C). The samples were stored at −20 °C before DNA was extracted. The DNA extraction protocol has been described previously by Horgan et al. (2017) [45] and is briefly described in the following section.

#### 2.3.2. DNA Extraction

A modified CTAB method adapted from Murray and Thompson (1980) [46] was used to extract DNA from the leaf samples. The young leaves were pulverized in a micro pestle using liquid nitrogen. After grinding, 50 µg of plant tissue was mixed with 750 µL of 2 × CTAB extraction buffer and 50 µL of 20% Sodium Dodecyl Sulfate (SDS). The resulting suspension was homogenized, incubated at 65 °C in a water bath for 30–60 min, and agitated every 15 min. The suspension was then cooled and an equal volume of chloroform: isoamyl alcohol (24:1) was added before thorough mixing and centrifugation at 14,000 rpm at 10 °C for 15 min. The upper aqueous phase was then transferred to a new 1.5 mL microtube and an equal volume of isopropanol was added, mixed thoroughly, and incubated overnight at −20 °C. On the following day, the suspension was centrifuged at 14,000 rpm at 4 °C for 15 min. The supernatant was decanted, the DNA pellet was twice washed with 500 µL of 70% ethanol and was air dried. The pellet was then dissolved in TE buffer of 100 µL + 1 µL of RNAse (Invitrogen™, Thermo Fisher Scientific, Carlsbad, CA, USA) (100 mg/mL) and incubated at 37 °C in a heat-block for 30 min. The DNA was then precipitated using 10 µL of 3 M sodium acetate and 200 µL of absolute ethanol and further incubated at −20 °C. The pellet was finally dissolved in 50 µL of TE buffer and quantified using 0.8% agarose gel and a NanoDrop 2000 UV-Vis Spectrophotometer.

#### 2.3.3. Genotyping Using 384-Plex SNP Set on the BeadXpress Platform

Genome-wide SNP genotyping was performed on the Illumina BeadXpress platform (Illumina, San Diego, CA, USA) using the *indica* × *indica* SNP set (GS0011861-OPA), which was designed to distinguish variation within the *indica* rice group (specifically, *indica* and *aus* subpopulations, *indica*/*indica* and *indica*/*aus* mapping populations) [47]. Scan results from the Illumina BeadXpress reader were initially analyzed using the Genotyping module (v1.6.3) of the Illumina GenomeStudio (v2010.1) to generate allele calls. The ALCHEMY algorithm [48] was further used to improve allele calls through the ALCHEMY-Illumina plug-in v1.0 in GenomeStudio.

### 2.4. Stemborer Antixenosis Experiments

We conducted choice and no-choice oviposition experiments with YSB. The protocol for the choice experiment has been described by Horgan et al. (2021) [34]. For the experiment, seedlings of each variety were transplanted at one seedling per hill to microplots in the screenhouse facility (described in Section 2.1). The microplots consisted of 32 plants (one for each variety) randomly positioned in one of six rows with a spacing of 35 × 35 cm between hills. Fertilizer was applied to the plots at a rate of 100 Kg N ha^−1^ 10 days after transplanting. The microplots were individually covered with mesh cages of 2.45 × 2.10 × 1.4 m (L × W × H). The entire experiment consisted of 6 replicated cages. At 20 DAS the cages were each infested with 40 gravid female YSB. After 5 days the adults were removed and the plants were carefully searched for egg masses. The number of egg masses per variety was noted and the masses were placed in scintillation vials until the larvae emerged, at which time the larvae were counted.

In a second, choice experiment, plants were grown in #6 pots as described above. At 30 DAS, the pots, one per variety, were randomly positioned in large cages (75 cm × 80 cm × 50 cm (H × L × W)) in a greenhouse (see conditions in Section 2.3.1) without foliage touching other plants or the cage walls. At 30 DAS, the cages were each infested with 70 gravid females. Cages were replicated six times, with each cage placed in a separate greenhouse compartment. After 5 days, the adults were removed and the plants were carefully searched for egg masses. The numbers of egg masses and eggs per variety were counted as described above.

### 2.5. Stemborer Antibiosis Experiments

Stemborer performance was assessed on the same plants in the microplots from the screenhouse choice experiment (see above: Horgan et al. (2021) [34]). At 35 DAT (after all the egg masses were removed from the microplots) each plant was infested with 10 neonate YSB. The neonates were placed on the plants above the water line using a fine paintbrush. Larvae were allowed to develop for 30 days before the plants were destructively sampled. During sampling, the plants were carefully pulled from the soil, were examined for larvae and pupae, and the number and condition of tillers were recorded. All collected YSB were counted and weighed. Each pupa was sexed and weighed separately. In total, we examined 192 plants (32 varieties × 6 replicates).

### 2.6. Planthopper Antixenosis Experiments

Plants were grown in pots: the rice seed was initially sown to saturated, homogenized paddy soil in plastic basins (25 × 30 × 50 cm, H × W × L) and, after 10 days, the seedlings were individually transplanted to size-0 pots (7 × 7 cm, H × D) with saturated paddy soil. The pots were placed in flooded trays to avoid heat stress and prevent interference by ants. The pots were watered daily and received no pesticide treatments. At 25 DAS, the pots, one per variety, were randomly positioned in large cages (75 cm × 80 cm × 50 cm (H × L × W)) in a greenhouse (see conditions in Section 2.3.1) ensuring that the foliage did not touch other plants or the cage walls. At 30 DAS, the cages were each infested with 70 gravid females. The experiment was conducted with plants grown under the zero-added fertilizer regime only; this is because nitrogen has a large impact on egg laying and using low and high nitrogen plants together in the experiment would otherwise obscure varietal preferences. Cages were replicated six times, with each cage placed in a separate greenhouse compartment. After 3 days, the positions of the adults were noted (i.e., settling) and the plants were destructively sampled, placed in plastic sleeves, and stored in a refrigerator (4 °C). All plants were searched for egg masses under a stereo microscope (×10 magnification) and the numbers of eggs per mass were counted.

### 2.7. Planthopper Antibiosis Experiments

Plants were grown in size 6 pots (15 × 15 cm, H × D) in a greenhouse (see conditions in Section 2.3.1). Half of the pots received fertilizer equivalent to 150 Kg N ha^−1^ by treating the soil with 0.124 g of ammonium sulfate one day before transplanting and a further 0.062 g at three days before infestation. This amount was based on the estimated weight of topsoil per hectare (2000 tons), the weight of soil in the size-6 pots (1.3 Kg pot^−1^), and the percentage of nitrogen contained in ammonium sulfate (21%). The remaining plants received no added fertilizer. At 25 DAS, the plants were each covered with an acetate cage (123 × 12 cm, H × D) with a nylon mesh window (23 × 15 cm: H × W) and nylon top.

At 30 DAS, plants were each infested with either two recently-emerged, gravid female BPH or two recently-emerged, gravid female WBPH. The herbivores were inserted into each cage through a slit in the acetate. The pots were arranged as a randomized replicated block design with six replicated blocks. Each block was located in a separate greenhouse compartment (separated by screened partitions) and consisted of 64 pots (32 lines × 2 herbivore treatments (BPH, WBPH) × 2 fertilizer treatments (0 and 150 Kg N ha^−1^)). The entire experiment consisted of 768 pots.

At 20 days after infestation (DAI), all planthoppers (BPH and WBPH) were removed from the plants using a vacuum sampler passed through the top of the cage (i.e., removing the top mesh). The cages were examined again after 7 and 14 days to collect any second-generation nymphs that developed from eggs already in the plants. These were added to the corresponding samples collected from each cage at 20 DAI. All collected insects were placed in glass test tubes and were immediately dried in forced draught ovens at 60 °C for one week. After drying, the samples were weighed (total weight per plant) and the developmental stages, sex, and condition (brachypterous or macropterous) of adults were recorded.

### 2.8. Field Plot Experiment

The occurrence of planthoppers, leafhoppers, and stemborers was recorded from field plots established at IRRI, Los Baños, Laguna, Philippines (14°11′ N, 121°15′ E). The plots were designed to examine factors contributing to yields, related to a separate study as described by Bueno and Lafarge (2017) [10].

The plots described in the present paper were planted during the wet season (June-October: Average temperature during early crop = 25.9 °C (22.6–29.2 °C, min-max); precipitation = 385 mm; relative humidity = 85%; average daily radiation = 15.6 MJ m^−2^) [10] when conditions are relatively unfavorable for rice herbivores, compared to the dry season. Briefly, the 32 varieties were laid out in a randomized complete block design with three replications. Pre-germinated seeds were first sown in seedling trays at 3000 seeds m^−2^. After 13 days, the seedlings were manually pulled and transplanted to the field at a hill spacing of 20 × 20 cm as one seedling per hill. The plot size was 50 m^2^. Phosphorus (40 Kg ha^−1^), potassium (20 Kg ha^−1^), and zinc (5 Kg ha^−1^) were incorporated into the plots on the day before rice transplanting. Nitrogen was applied to the plots at a rate of 40 Kg ha^−1^ seven days after transplanting; this was the first of four splits to attain 150 Kg ha^−1^ for the entire crop [10]. The field was flooded 3 days after transplanting and had 3–5 cm of standing water throughout the course of the observations.

Pests were extensively controlled in the plots using chemicals; however, applications were avoided until 40 days after transplanting during the wet season. Therefore, we visually sampled the plots at 35 DAT, before any pesticides had been applied. Sampling was conducted by randomly selecting six hills per plot and carefully examining the plants for the presence of planthopper adults and nymphs. We recorded BPH, WBPH, and leafhoppers (*Nephotettix* spp.) only. For stemborers, we randomly selected 50 hills and examined plants for symptoms of deadheart. The affected tillers were carefully pulled to identify the stemborer species as YSB or striped stemborer (*Chilo suppressalis* (Walker) SSB). All sampled hills were located at the centers of the plots, avoiding the outer four rows of plants in each plot. To avoid chemical pest management measures conducted in the plots, the insects were recorded during only one sampling event (about maximum tillering).

### 2.9. Data Analyses

This study recorded a large amount of data related to 32 rice genotypes. The main hypotheses for the study relate to plant type and the origin of genotypes; therefore, we present our results in the main text for plant type based on the averages for 20 hybrids and 12 inbreds and, where relevant, included fertilizer levels. We further explored trends across the 32 genotypes based on plant type and origin using multivariate analyses (see below) to improve inferences. We have also included the results for each of the genotypes and all experiments in a series of Supplementary Tables.

We used univariate General Linear Models (GLM) to compare tillering, shoot, and root parameters and SPAD values across all genotypes (i.e., accession = 32 levels) based on screenhouse data (main factors = accession and fertilizer level). The same analyses were conducted with BPH and WBPH fitness parameters (proportion as adults, females, and brachypterous adults, population size, and total planthopper biomass). Planthopper and stemborer oviposition responses and stemborer fitness responses from the screenhouse study were analyzed using univariate GLMs. The factor ‘block’ was initially included in analyses for greenhouse and screenhouse data, but was not significant and was subsequently removed. Models initially included tiller numbers and plant biomass as covariates, but these were removed when non-significant. YSB pupal weights were analyzed using a two-way analysis with accession and sex as the main factors. Field data was analyzed using multivariate GLMs for planthopper species and univariate GLM for deadhearts (i.e., YSB damage). The data were then reanalyzed with plant types (two levels) and, where available, fertilizer levels (two levels) using univariate GLMs (as reported in the main text). Pairwise comparisons were made using Tukey’s LSD tests. All settling and oviposition data were ranked within replicates before analyses to meet the requirement for the independence of samples. Biomass and population size data were log(x + 1)-transformed before all analyses; proportion data were arcsine-transformed before analyses. Residuals were plotted after each analysis to check that they were normal and homogenous.

We used Spearman’s rank correlations to assess the correspondence between fitness parameters recorded from the same experiments, parameters from oviposition and fitness bioassays for each species, and between parameters from screenhouse or greenhouse environments and results from the field plots. We use multiple regression with backward elimination to determine the best predictors (from measured plant parameters) of stemborer survival and biomass from the screenhouse study.

Permutational multivariate analysis of variance (PERMANOVA) [49] was used to test for (i) genetic differences (based on SNP genotyping) between genotypes, and (ii) differences across resistance-related variables to BPH, WBPH, and YSB of rice genotypes. We included two factors in each analysis: ‘plant type’ (fixed factor) had two levels (hybrid and inbred) and; ‘origin’ (fixed factor) had four levels (Philippines, India, Colombia, and China). PERMANOVA analyses were based on Bray-Curtis similarity resemblance matrices of root square-transformed data. Each analysis was permutated 9999 times. We used Canonical Analysis of Principal Coordinates (CAP) to visualize the differences between resistance-related variables across factors [50]. Each CAP ordination analysis was calculated based on significant results obtained with PERMANOVA. PRIMER (v. 6.1.16) with the PERMANOVA + extension (v. 1.0.6) was used to perform the PERMANOVA and CAP routines. To determine genetic differentiation between rice varieties belonging to different plant types (hybrid/inbred) and different geographical origins, 321 SNPs were used (≥90% calling; ≥0.05% minor allele frequencies) for computation of pairwise [51] and hierarchical F_st_ [52] similarities using Hierfstat v. version 0.5–11 in R [53].

## 3. Results

### 3.1. Plant Development and Genotyping

Nitrogen increased tiller numbers, plant height, plant weights, and SPAD readings (Table S2) for all plants. There was a significant accession × nitrogen effect on tillering in the screenhouse facility; this was due to a relatively low increase in tillering of H35, H36, and I45 compared to the other genotypes under the high nitrogen regime (Table S2). The hybrids H1, H3, H9, H17, H32, and H35 had the highest rates of tillering. There were significant accession × nitrogen interactions for root and shoot biomass because of relatively poor responses to nitrogen in several accessions (roots: H17, H20, H31, H35, H36, I1, I2, I4, I7, I40, I41, I42, I45; shoots: H19, H31, H36, I1, I2, I4, I7, I41 and I42: Table S2). I43 and I46 had the heaviest roots (Table S2) and were significantly heavier than eight of the other inbreds and five of the hybrids. H16 had the heaviest shoots, and these were heavier than two inbreds (I41, I42) and two hybrids (H19, H36) (Table S2). For the full details of plant growth parameters, see Table S2.

**Figure 1:**
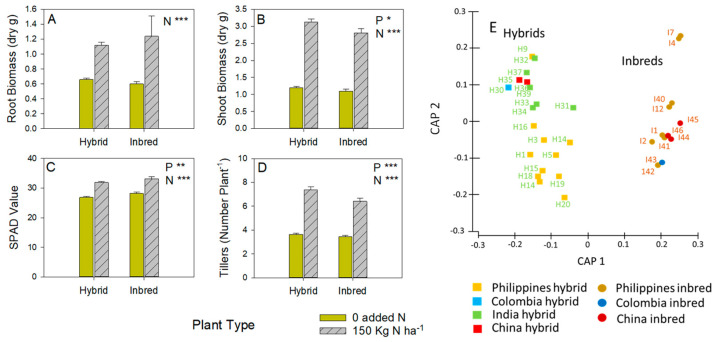
(**A**) Root dry weight (biomass), (**B**) shoot biomass, (**C**) SPAD values, and (**D**) tiller numbers of hybrid and inbred varieties grown in a screenhouse environment and harvested at early tillering (35 DAS). Results of univariate GLMs are indicated with each graph as P = plant type and N = nitrogen with * = *p* ≤ 0.05, ** = *p* ≤ 0.01, and *** = *p* ≤ 0.005. Means and standard errors (N = 12–20, see Table S1) are shown. Full details of growth parameters for each genotype are presented in Table S2. (**E**) Canonical Analysis of Principal Coordinates (CAP) showing clustering (based on SNP genotyping) of rice genotypes, by plant type (squares and lighter shading = hybrids; circles and darker shading = inbreds) and origin (yellow = Philippines; blue = Colombia, green = India; red = China).

Among the selected genotypes, hybrids and inbreds had similar root weights (Figure 1A), and the hybrids produced larger shoots (F_1,60_ = 6.908: Figure 1B) with lower SPAD values (F_1,60_ = 7.831: Figure 1C). Hybrids produced significantly more tillers (F_1,60_ = 8.979: Figure 1D). Nitrogen increased all growth parameters (F_1,60_ > 25.000) without significant interactions (Figure 1).

PERMANOVA tests of genetic characterization across genotypes based on SNPs indicated a significant differentiation between hybrid and inbred varieties (Pseudo-F = 4.858, *p* = 0.001) but not between geographical origins (Pseudo-F = 0.764, *p* = 0.868) (Figure 1E). There was moderate genetic differentiation (F_st_ = 0.07) between hybrid and inbred varieties but no genetic differentiation (F_st_ < 0.045) between varieties when grouped based on different geographical origins. Plant type and geographical origins had no significant effect (*p* ≥ 0.17) on the genetic differentiation of the 32 rice varieties in this study.

### 3.2. Stemborer Responses to Hybrid and Inbred Lines

The was no significant effect of genotype on stemborer oviposition in the greenhouse study (Table S3). In the screenhouse, very few eggs were laid on I46 and significantly fewer than on H19 (Table S4). Few eggs were laid on I46 in the greenhouse choice experiment also; however, egg laying in the two experiments was poorly correlated (rs_21_ = −0.168, *p* = 0.468). Stemborer survival and biomass were significantly lower on I45 compared to H3; across accessions, tiller numbers significantly influenced the numbers and biomass of survivors (Table S4). The proportions of dead tillers (deadheart) were relatively low on H18 and I45, and high on H9 and H31 (Table S4). The proportion of deadheart was significantly correlated with the numbers (rs_192_ = 0.396) and biomass (rs_192_ = 0.249) of stemborers. Plant biomass was the best predictor of both stemborer survival and final YSB biomass (survivors: F_1,95_ = 4.429, *p* = 0.04; biomass: F_1,95_ = 4.856, *p* = 0.03).

**Figure 2:**
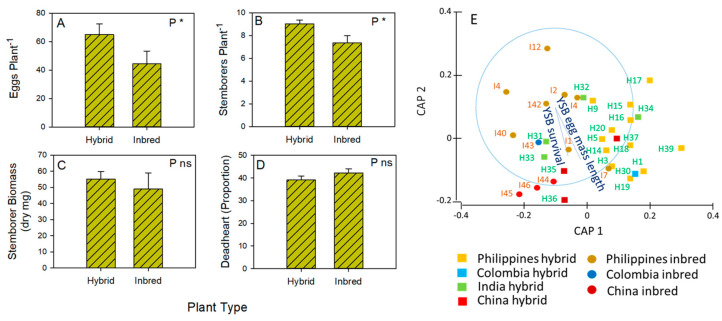
(**A**) Stemborer eggs per plant from a choice microplot experiment with (**B**) the number of surviving stemborers (from a maximum of 10), (**C**) stemborer biomass, and (**D**) the proportion of tillers that were damaged. Results of univariate GLMs are indicated with each graph as P = plant type with ns = *p* > 0.05 and * = *p* ≤ 0.05. Means and standard errors (N = 12–20) are shown. Full details are presented in Table S4. (**E**) Canonical Analysis of Principal Coordinates (CAP) indicating responses by YSB to rice genotypes as a function of plant type (squares and lighter shading = hybrids; circles and darker shading = inbreds) and origin (yellow = Philippines; blue = Colombia, green = India; red = China). Overlayed vectors (in blue) represent correlations (Pearson’s correlation coefficient > 0.6) where vector length and direction reflect the increasing values of correlation and parameter values, respectively.

Among the selected genotypes, egg laying (F_1,60_ = 6.371: Figure 2A) and larval survival (F_1,60_ = 7.172: Figure 2B) were greater on hybrids than inbreds, but there were no plant type effects on final stemborer biomass (F_1,60_ = 0.319: Figure 2C) or the proportion of dead hearts (F_1,60_ = 1.346: Figure 2D). PERMANOVA test results showed significantly different responses by YSB to hybrid and inbred genotypes (Pseudo-F = 1.899, *p* = 0.048) (Figure 2E). Correlation tests indicated the highest YSB survival (i.e., the number of larvae per plant) on the Chinese hybrid and the lowest on Philippine inbred genotypes.

### 3.3. Planthopper Responses to Hybrid and Inbred Lines

Significantly more BPH settled on H30 than on H37, H39, I41, and I44; more egg masses and eggs were laid on H30 than on I7 (Table S5). The numbers of egg masses and eggs were correlated with plant weight for BPH (egg masses: N = 30, rs_32_ = 0.469, *p* = 0.009; eggs: N = 30, rs_32_ = 0.483, *p* = 0.007). In terms of biomass build-up, I42 appeared significantly more resistance to BPH than H1, H5, H9, H14, H15, H18, H31, H33, H36, H39, I4, I7, I12, I40, I44 and I45. For other fitness parameters see Table S6. BPH numbers and biomass were correlated on low (rs_32_ = 0.702, *p* < 0.001) and high (rs_32_ = 0.481, *p* < 0.005) nitrogen, but were not correlated across nitrogen levels or with settling and oviposition.

**Figure 3:**
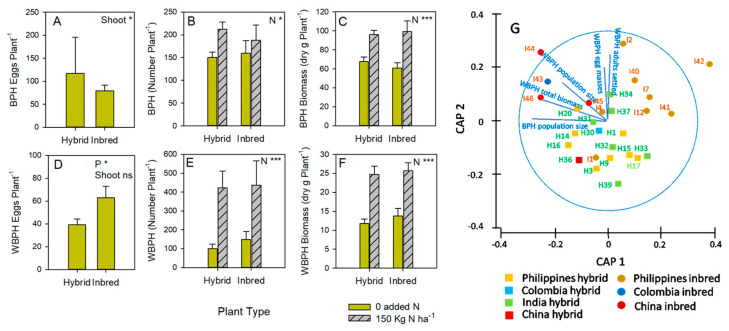
(**A**) BPH eggs per plant in a greenhouse choice experiment with (**B**) the number of BPH per plant (after infestation with two gravid females) and (**C**) the biomass of BPH on the same plants. Results for (**D**) WBPH eggs per plant from a greenhouse choice experiment, (**E**) the number of WBPH per plant (after infestation with two gravid females), and (**F**) the biomass of WBPH on the same plants are also presented. The results of univariate GLMs are indicated with each graph as P = plant type, N = nitrogen, and ‘Shoot’ = covariate shoot biomass with ns = *p* > 0.05, * = *p* ≤ 0.05, and *** = *p* ≤ 0.005. Means and standard errors (N = 12–20) are shown. Note that the choice oviposition preference experiment had plants under zero-added nitrogen only. Full details are presented in Tables S6 and S7. (**G**) Canonical Analysis of Principal Coordinates (CAP) showing differences in responses by planthoppers to rice genotypes as a function of plant type (squares and lighter shading = hybrids; circles and darker shading = inbreds) and origin (yellow = Philippines; blue = Colombia, green = India; red = China). Overlayed vectors (in blue) represent the correlations (Pearson’s correlation coefficient > 0.6) between resistance-related variables and the CAP axes, where vector length and direction reflect the increasing values of correlation and parameter values, respectively.

Across the genotypes, there was no significant effect of plant type on egg laying by BPH (F_1,27_ = 0.068); the covariate shoot biomass had a significant effect on BPH egg-laying (F_1,27_ = 4.451: Figure 3A). Nitrogen affected the numbers (F_1,60_ = 4.656: Figure 3B) and biomass (F_1,60_ = 26.569: Figure 3C) of BPH at the end of the greenhouse experiment; plant type had no effect on the numbers (F_1,60_ = 0.114) or biomass (F_1,60_ = 0.074) (Figure 3).

Significantly more WBPH adults settled on I2 and I42 than on H15, H6, H17, and H33; more egg masses and eggs were laid on I2 than on H1, H15, H17, and H33 (Table S5). I44, I45 and H36 were the most susceptible genotypes, with H17, I42 and I42 relatively resistant (Table S7). WBPH numbers and biomass were correlated for low (rs_32_ = 0.773, *p* < 0.001) and high (rs_32_ = 0.489, *p* = 0.004) nitrogen, and across nitrogen levels (numbers: rs_32_ = 0.723, *p* < 0.001; biomass: rs_32_ = 0.490, *p* = 0.004). Furthermore, settling and egg laying by WBPH were correlated with numbers (settling: rs_28_ = 0.439, *p* = 0.019; eggs: rs_28_ = 0.474, *p* = 0.011) and biomass (settling: rs_28_ = 0.404, *p* = 0.033; eggs: rs_28_ = 0.391, *p* = 0.040) in the greenhouse study.

Across the genotypes, WBPH laid more eggs on the inbred varieties (F_1,27_ = 6.371: Figure 3D); the covariate shoot biomass had no significant effect on WBPH egg-laying (F_1,27_ = 1.733). Nitrogen affected the numbers (F_1,60_ = 15.222: Figure 3E) and biomass (F_1,60_ = 35.808: Figure 3F) of WBPH on the plants without plant type effects (numbers: F_1,60_ = 0.164; biomass: F_1,60_ = 0.350) (Figure 3).

Planthoppers (BPH and WBPH) showed a significant response to plant types, as indicated by the PERMANOVA test results (Pseudo-F = 3.077, *p* = 0.012: Figure 3G). This effect was shown on the CAP ordination plot, with most hybrid varieties located at the bottom of the plot and inbred varieties at the top (Figure 3G). Correlation analyses (based on Pearson’s coefficient < 0.6) indicate WBPH settling and egg laying responses were largely determined by Philippines inbreds and I45, with relatively large population size and biomass responses by WBPH (particularly) and BPH to the Chinese inbreds (Figure 3G).

### 3.4. Pest Occurrence in Field Plots

WBPH was the most frequently observed planthopper species in the field plots. H17 was more resistant than H20, H30, and I45 to the planthopper. There was no accession effect on BPH and GLH occurrence (Table S8). SSB was more common in the field plots than YSB: deadhearts were more prevalent in plots of I44 and least prevalent in H15 and H33 (Table S8).

**Figure 4:**
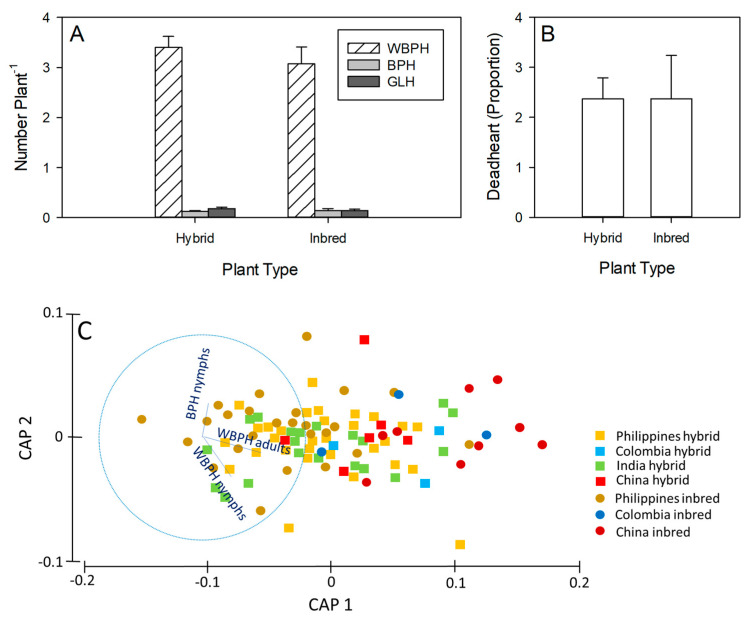
Results from sampling of insect herbivores in field plots at 35 DAS indicating (**A**) the densities of planthoppers and leafhoppers per plant, and (**B**) the proportion of tillers that were attacked by stemborers. Means and standard errors (N = 12–20) are shown. For further details see Table S8. (**C**) Canonical Analysis of Principal Coordinates (CAP) showing differences in responses by BPH, WBPH, and YSB to the rice genotypes as a function of plant type (squares and lighter shading = hybrids; circles and darker shading = inbreds) and origin (yellow = Philippines; blue = Colombia, green = India; red = China). Overlayed vectors (in blue) represent the correlations (Pearson’s correlation coefficient > 0.6) between resistance-related variables and the CAP axes, where vector length and direction reflect the increasing values of correlation and parameter values, respectively.

Across genotypes, there was no effect of plant type on WBPH (F_1,30_ = 0.630), BPH (F_1,30_ = 0.002), GLH (F_1,30_ = 1.180) (Figure 4A), or deadhearts (F_1,30_ = 0.430) (Figure 4B). Origin had a large effect (α = 0.1) on pest infestations (represented by the CAP 1-axis) (Pseudo-F = 1.5513, *p* = 0.077) with field plots of Chinese inbreds having the highest pest densities (Figure 4C).

## 4. Discussion

### 4.1. Hybrid and Inbred Rice Genotypes

In our experiments, the hybrids had generally higher shoot biomass and higher tillering than the inbreds at the same growth stage (Figure 1B,D). The differences in hybrid shoot biomass and tillering were more apparent between hybrids and inbreds under higher fertilizer conditions, indicating the greater responses by hybrids to available soil nitrogen [19]. Such faster growth rates contribute to the potential yields of hybrids. In a parallel study, the average yield of the hybrids exceeded that of the inbreds by about 0.6 t ha^−1^ (about 7%) during the dry season; however, inbreds had higher average yields (about 0.3 t ha^−1^; about 5%) than the hybrids during the wet season [10] (Table S1). Faster growth rates and hybrid plant anatomy have also been shown to influence hybrid susceptibility to herbivores, particularly leaffolders and stemborers [14,19].

Our CAP plot, based on an analysis of SNP markers, showed a clear separation of the hybrid from the inbred genotypes (Figure 1E). Furthermore, the hybrids had two prominent clusters largely representing Philippines hybrids and others (China, Colombia, and India). We expected that some of the clustering might indicate closer relations between genotypes from the same countries irrespective of plant type. That this did not occur demonstrates the large effect that male-sterile lines have on hybrid diversity [5,54]. A majority of hybrid rice varieties still rely on lines derived from the original WA-CMS genotype in 3-line breeding systems [5,6,11,15,55]. This dependency on a restricted group of female parental lines, and further limitations on the availability of lines capable of restoring fertility [2], likely creates a diversity bottleneck for hybrids. Several closely related genotypes (e.g., I2 and I7, I44 and I46, H35 and H36: Figure 1E) were from the same breeding programs; however, similar genotypes from different regions (e.g., H9 and H32) or from different breeding programs in the same region (e.g., H33 and H34) indicate the probable effects of sharing breeding materials between programs. Indeed, some of the genotypes we used were sourced through the Hybrid Rice Development Consortium (HRDC), which aims to strengthen collaboration between public and private sectors and improve the dissemination of hybrid technologies [6]. Furthermore, some of the materials, particularly the Chinese hybrid and Inbred lines, were elite lines from the Green Super Rice (GSR) Project that aims to develop and deploy new high-yielding varieties that require fewer inputs (e.g., fertilizers, pesticides, etc.) for lower- and middle-income countries [56,57].

The clustering of the genotypes into a hybrid and an inbred group limited our ability to separate plant type effects from phylogenetic effects on herbivore fitness responses; however, by including plant origin in our analyses, we revealed a number of breeding-related issues that influence host susceptibility/resistance to the herbivores. The effects of plant type and origin on herbivore responses to rice are discussed in the following sections.

### 4.2. Stemborer Responses to Hybrid and Inbred Genotypes

Several studies have reported hybrid rice varieties as more susceptible than inbred varieties to stemborers [5,11,14,18,58,59]. This includes studies that compared stemborer fitness and damage on arbitrarily selected or most commonly planted hybrid and inbred varieties [5,18], as well as studies that compared fitness responses to hybrids and their inbred parental lines [11,14,19]. The underlying susceptibility of hybrids has mainly been attributed to plant physiology and anatomy. In particular, the relatively high tillering by hybrids and thick stems are attractive to some stemborer species, including both YSB and SSB [34]; furthermore, faster growth rates and the relatively greater biomass accumulation by hybrids increase the fitness of stemborers on some hybrids compared to inbreds [5,11]. Varieties with relatively long duration are also more vulnerable to stemborer attacks [11,34]. In our experiments, YSB laid more eggs on hybrid varieties and larval survival was higher on the hybrids compared to inbreds (Figure 2A,B). Plant biomass was the best predictor of stemborer fitness, and the hybrids had generally higher shoot biomass compared to inbreds (Figure 1).

The PERMANOVA results further indicated that susceptibility can be largely attributed to the hybrid plant type (Figure 2E). The results were strongly affected by relatively high stemborer fitness (survival, biomass, development, proportion of deadhearts, and eggs laid) on a number of hybrids (indicated by the CAP 1-axis in Figure 2E). Among the most susceptible genotypes were H30 (Colombia), H35 and H36 (China), and I2 and H3 (the Philippines). These results therefore support previous studies to suggest that hybrids are generally more susceptible to stemborers. However, some inbreds (including the closely related I2 and I7 genotypes) are as susceptible to stemborers as are hybrids; furthermore, H1, H5, H16, H18, and H32 were among the least susceptible genotypes in the screenhouse study. Therefore, although hybrids tend to be more susceptible, high-yielding hybrids with relatively low susceptibility to stemborers can be achieved.

YSB counts, SSB counts and proportions of dead tillers (deadhearts) recorded from the field plots were well correlated (rs_32_ > 0.409, *p* < 0.02). However, damage and stemborer counts were not generally related to the estimated fitness parameters from the screenhouse experiments (rs_32_ < 0.346). Damage in the field is determined by stemborer abundance and behavior and is influenced by mortality due to natural enemies and interactions with competitors [34], which may have obscured the direct effects of the host plants. Despite a lack of significant correlations, the most susceptible genotypes in the field plots still included H30 and H35, which were associated with high stemborer survival in the screenhouse, and I44, which was associated with high numbers of emerging larvae in the screenhouse choice experiment. Among the least susceptible genotypes in the field, only I1 was also associated with relatively low stemborer fitness in the screenhouse experiments (Table S4). Results from the field plots therefore indicated a number of genotypes with high susceptibility to stemborers that should be avoided during further breeding selection.

### 4.3. Planthopper Responses to Hybrid and Inbred Genotypes

Our results largely corroborate recent findings that female-derived hybrid susceptibility to planthoppers, which was relatively high in previous studies [5], has since declined: we found no plant-type effects on antibiosis responses by WBPH or BPH (Figure 3B–F). WBPH egg-laying was generally higher in inbred genotypes (Figure 3G). The most susceptible genotypes from the antibiosis experiments included hybrids and inbreds from China (e.g., H36, I44, I45, and I46), Colombia (I43), and India (H31 and H34), with the least susceptible genotypes including hybrids and inbreds from the Philippines (H17, I41 and I42) and India (H32 and H39) (Figure 3G). Under high nitrogen conditions, H31, H36, I44, and I45 were also relatively susceptible to one or both planthoppers (i.e., susceptibility was consistent under low and high nitrogen). Overall, the closely related Chinese inbred lines (Figure 1E) and I43 appeared consistently susceptible to both BPH and WBPH based on several fitness parameters (Tables S6 and S7).

In field plots, BPH and *Nephotettix* spp. numbers were highly correlated (rs_32_ = 0.567, *p* < 0.001); however, the numbers were relatively low (Table S8). Despite BPH facilitating WBPH attacks [60,61], the numbers of these two planthopper species were not correlated across genotypes in the field. WBPH numbers from the field plots were highly correlated with the numbers and biomass of WBPH on greenhouse plants under high (numbers: rs_32_ = 0.566, *p* < 0.001; biomass: rs_32_ = 0.584, *p* < 0.001) and low (numbers rs_32_ = 0.441, *p* = 0.011; biomass: rs_32_ = 0.462, *p* = 0.008) nitrogen. Furthermore, the BPH counts from the field plots were correlated with the biomass of BPH under equivalent high nitrogen in the greenhouse study (rs_32_ = 0.346, *p* = 0.052). Such close correlations between the results of greenhouse and field studies are unusual because natural enemies can obscure the effects of host plant resistance on planthopper numbers in field studies [33,40,60,61]. By sampling early in the season, we may have avoided the confounding effects of field mortalities and thereby gained confidence in our results for planthopper responses to the genotypes. In agreement with the greenhouse study, the Chinese inbred genotypes I44, I45, and I46, together with H20 (Philippines) and H30 (Colombia), had the highest densities of WBPH. Our results therefore strongly indicate that the origin of the genotypes (i.e., the country or breeding programs) in our study had a greater effect than plant type on rice susceptibility to planthoppers.

### 4.4. Recommendations

Greater attention to rice anti-herbivore resistance can reduce the current high levels of pesticide use that are apparent throughout much of tropical Asia [62,63,64]. There are three main approaches by which breeding programs can reduce rice susceptibility to herbivores. Firstly, programs can ensure that the most susceptible lines are eliminated from breeding after screening early in the breeding pipeline [45,65]. Secondly, programs can increase the genetic distance between varieties, because genetic distance and host novelty represent a barrier to herbivory, especially for planthoppers [66,67]. Thirdly, programs can introduce resistance from diverse sources (including traditional varieties and landraces or wild rice species) through marker-assisted breeding [68,69,70,71,72,73].

Susceptible genotypes may be inadvertently maintained in breeding programs because of insufficient screening during varietal development or because breeders accept susceptibility trade-offs in favor of higher yields or other desirable plant traits [74]. It is also possible that genotype × environment interactions may have increased the susceptibility of some imported lines under the warm conditions of the Philippines wet season [75,76,77]. Rice lines that appeared relatively resistant to the herbivores, and particularly to stemborers, in our study, represent valuable materials for breeding programs, especially since any resistance was likely due to quantitative traits. We recommend that breeding programs develop varieties with good quantitative resistance even if they possess major resistance genes because this increases resistance durability [78,79]. Furthermore, although we did not address tolerance in our study, we recommend that future research include tolerance (the plant’s ability to compensate for damage) [37,80,81,82] together with at least moderate resistance [19,83] for sustainable planthopper and stemborer management.

Contrary to expectations (based on herbivore naivety), many of the most susceptible genotypes in our study were from China and Colombia. These imported varieties did not represent a barrier to naïve herbivores from the Philippines. This may be attributed to their close genetic relations with the Philippines genotypes (most of which originated from IRRI) (Figure 2E), possibly through the widescale sharing of breeding materials [84,85]. By increasing the diversity of materials in breeding programs, the genetic similarity of varieties might be addressed. This is particularly relevant to hybrid lines that are funneled through 3-line breeding systems (Figure 2E). By incorporating diverse breeding lines, the HRDC and GSR programs have already begun to rectify the hybrid diversity issue.

## 5. Conclusions

Based on the results of our study, the hybrid plant type was associated with susceptibility to YSB. Greater tillering and higher growth rates that resulted in relatively large plants were more attractive to ovipositing females and allowed a higher survival of larvae. However, the results from the screenhouse and field were not correlated and, in the field plots, several hybrids were relatively resistant to stemborers, whereas certain inbreds were among the most susceptible genotypes. Therefore, whereas hybrids are prone to be susceptible to stemborers, relatively stemborer-resistant high-yielding hybrid varieties are possible. Our results did not support the hypothesis that the hybrid plant type is more susceptible to planthoppers. The origin of the genotypes (varieties) largely determined whether plants were susceptible to BPH or WBPH. High-yielding hybrids with relatively good resistance to all three herbivores were identified. Resistance in these hybrid genotypes was not associated with any major resistance genes but was probably due to quantitative traits.

## Data Availability

The data presented in this study are available upon reasonable request from the corresponding author.

## Supplementary Materials

The following supporting information can be downloaded at: https://www.mdpi.com/article/10.3390/insects15080608/s1, Figure S1: Schematic diagram summarizing the bioassays and data collected in the present study, Table S1: Genotypes used in the experiments with information on crop duration, tillering and grain yields, Table S2: Growth parameters for 32 rice genotypes at 35 days after sowing under low and high nitrogen conditions, Table S3: Results from stemborer preference (antixenosis) and performance (antibiosis) experiments conducted in a screenhouse environment, Table S4: Results from greenhouse oviposition (performance) experiment with yellow stemborer, Table S5: Results of adult settling and oviposition (preference) experiments with brown planthopper (BPH) and whitebacked planthopper (WBPH) in greenhouse cages, Table S6: Performance (population build-up) of brown planthoppers (BPH) on 32 rice genotypes in a greenhouse experiment, Table S7: Performance (population build-up) of whitebacked planthoppers (WBPH) on 32 rice genotypes in a greenhouse experiment, Table S8: Results from monitoring of field plots at 35 DAS.
