# Supplementary material for: Origins of Susceptibility to Insect Herbivores in High-Yielding Hybrid and Inbred Rice Genotypes
Source: Insects. 2024 Aug 12;15(8):608. doi: 10.3390/insects15080608 (PMC11354228; doi:10.3390/insects15080608)
Supplement: Supplementary file 1 [file insects-15-00608-s001.zip › insects-3110672-supplementary.pdf]

Supplementary Information

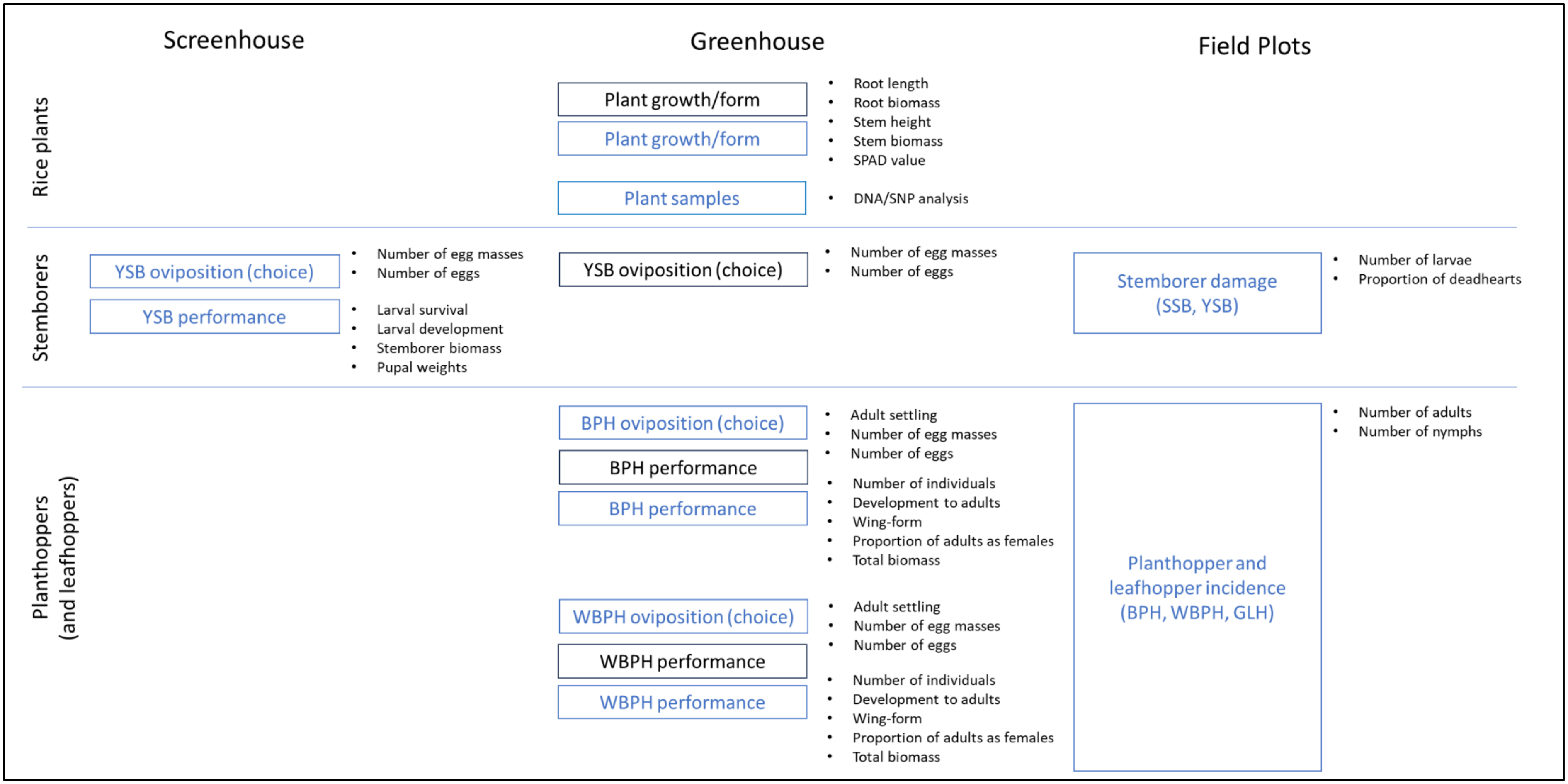

Figure S1. Schematic diagram summarizing the bioassays and data collected in the present study. Black rectangles indicate low nitrogen conditions, blue rectangles indicate high (100-150 Kg ha-1) nitrogen conditions in protected and field environments. All bioassays were replicated six times, except the field study that had three replicates for each genotype. The experiments were based on 20 hybrid and 12 inbred lines as listed in Table S1. YSB = yellow stemborer, SSB = striped stemborer, BPH = brown planthopper, WBPH = whitebacked planthopper, and GLH = green leafhopper. Full details of each bioassay are presented in the main text.

Table S1. Genotypes used in the experiments with information on crop duration, tillering and grain yields during the wet and dry seasons as reported by Bueno and Lafarge (2017)[1]

Genotype Codes	Plant Type	Origin	Crop duration during the Dry Season (Days)	Crop duration during the Wet Season (Days)	Maximum tillering during the Dry Season (Number of Tillers)	Dry Season Yields (Tons ha^{-1})	Wet Season Yields (Tons ha^{-1})
H1	Hybrid	Philippines	105	109	672	9.38	7.06
H3	Hybrid	Philippines	111	115	542	9.52	4.77
H5	Hybrid	Philippines	105	111	719	9.46	7.63
H9	Hybrid	Philippines	113	118	656	9.31	5.68
H14	Hybrid	Philippines	105	111	690	9.63	7.38
H15	Hybrid	Philippines	105	115	634	9.59	6.50
H16	Hybrid	Philippines	105	109	693	9.46	8.73
H17	Hybrid	Philippines	105	111	723	9.44	6.07
H18	Hybrid	Philippines	107	111	515	9.50	6.89
H19	Hybrid	Philippines	107	118	693	9.38	5.57
H20	Hybrid	Philippines	117	115	651	8.72	7.09
H30	Hybrid	Colombia	105	111	555	9.77	7.25
H31	Hybrid	India	107	118	622	9.39	6.90
H32	Hybrid	India	113	118	695	9.30	5.96
H33	Hybrid	India	105	115	775	9.36	6.64
H34	Hybrid	India	113	118	640	8.22	5.27
H35	Hybrid	China	107	115	554	9.24	6.57
H36	Hybrid	China	111	115	481	8.56	6.94
H37	Hybrid	India	113	111	552	8.15	6.34
H39	Hybrid	India	117	118	723	9.04	5.87
I1	Inbred	Philippines	111	111	795	8.89	7.07
I2	Inbred	Philippines	105	109	879	8.53	6.91
I4	Inbred	Philippines	111	111	590	9.08	7.15
I7	Inbred	Philippines	113	122	704	9.18	6.79
I12	Inbred	Philippines	111	115	560	8.89	6.43
I40	Inbred	Philippines	107	118	750	8.44	7.77
I41	Inbred	Philippines	107	115	668	8.30	7.40
I42	Inbred	Philippines	107	115	582	8.52	6.92
I43	Inbred	Colombia	117	122	513	7.45	5.39
I44	Inbred	China	111	111	426	8.81	8.18

I45	Inbred	China	105	109	272	7.79	5.80
I46	Inbred	China	111	115	544	9.06	7.86

1: Bueno, C.S.; Lafarge, T. Maturity groups and growing seasons as key sources of variation to consider within breeding programs for high yielding rice in the tropics. *Euphytica* **2017**, *213*, 1-18.

Table S2. Growth parameters for 32 rice genotypes at 35 days after sowing under low and high nitrogen conditions in a screenhouse experiment at the International Rice Research Institute.

Genotypes	Applied Nitrogen (Kg ha^{-1})	Number of Tillers[1]	Root Length (cm)[1]	Root Biomass (Dry g)[1]	Shoot Height (cm)[1]	Shoot Biomass (Dry g)[1]	Total Plant Biomass (Dry g)[1]	SPAD Values[1]
H1	0	4.33±0.21e	30.00±1.34ab	0.64±0.03ab	53.58±1.27defgh	1.36±0.07abc	2.00±0.09ab	27.45±0.61abc
	40	9.50±0.92	28.42±1.57	1.27±0.18	63.83±0.95	3.47±0.48	4.74±0.09	30.50±0.42
H3	0	3.33±0.21bcde	31.08±1.78ab	0.67±0.08ab	50.50±1.26abcdefg	1.12±0.08abc	1.80±1.09ab	27.77±0.56abcde
	40	8.00±0.45	31.00±1.12	1.32±0.12	61.25±0.86	3.01±0.21	4.32±1.09	32.37±0.12
H5	0	3.50±0.22de	28.50±1.94a	0.70±0.17ab	50.75±1.01abcdef	0.99±0.16abc	1.70±0.09ab	27.82±1.22abcd
	40	8.33±0.42	29.50±1.26	1.52±0.20	58.50±0.67	3.43±0.22	4.94±0.09	31.53±0.38
H9	0	3.33±0.21de	27.42±1.11a	0.73±0.03ab	49.01±0.76abcd	1.11±0.08abc	1.84±0.09ab	25.17±1.14a
	40	8.83±1.19	29.83±1.72	1.24±0.11	58.50±0.66	3.34±0.34	4.58±0.09	29.43±0.62
H14	0	3.83±0.17bcd	29.33±0.81ab	0.63±0.03ab	49.08±1.16abcdefg	1.22±0.10abc	1.86±0.09ab	27.05±0.48abcde
	40	6.50±0.43	29.83±0.71	1.15±0.10	61.00±0.87	3.14±0.12	4.29±0.09	32.57±0.45
H15	0	3.17±0.40bcde	29.00±1.09ab	0.50±0.06ab	54.00±1.48defgh	1.06±0.15abc	1.56±0.09ab	28.35±1.26abcd
	40	8.00±0.45	29.50±0.99	1.15±0.11	64.00±1.28	3.33±0.42	4.48±0.09	30.73±0.56
H16	0	3.50±0.22bcde	34.67±1.63ab	0.66±0.12ab	55.50±0.79fgh	1.42±0.17c	2.08±0.09b	27.73±0.50abcde
	40	7.33±0.67	28.93±1.32	1.17±0.12	64.33±1.84	3.85±0.22	5.03±0.09	32.58±0.54
H17	0	3.83±0.31de	30.50±1.09ab	0.54±0.07a	48.83±2.01abcd	1.13±0.13abc	1.67±0.09ab	27.58±1.26abcde
	40	8.50±0.34	31.08±1.93	0.98±0.09	59.33±0.98	2.90±0.26	3.88±0.09	32.48±0.82
H18	0	3.33±0.21bcd	29.67±1.31ab	0.70±0.06ab	47.08±2.68abc	1.25±0.11abc	1.95±0.09ab	27.05±0.63abcde
	40	7.00±0.97	30.92±1.67	1.19±0.11	59.33±1.03	3.26±0.20	4.44±0.09	33.67±1.05
H19	0	3.40±0.24abcd	29.90±2.68ab	0.69±0.10ab	53.00±1.19cdefgh	1.09±0.12ab	1.48±0.09ab	25.72±0.67ab
	40	6.00±0.52	30.40±1.65	1.02±0.15	61.80±1.40	2.28±0.32	3.31±0.09	29.40±0.58
H20	0	3.67±0.49cde	28.92±1.66ab	0.66±0.08a	52.00±1.72cdefgh	1.21±0.15abc	1.86±0.09ab	28.28±1.10abcde
	40	8.00±0.89	33.75±1.59	0.92±0.08	65.00±1.35	3.20±0.40	4.12±0.09	32.40±0.84
H30	0	3.83±0.40bcd	29.50±2.00ab	0.77±0.12ab	54.92±1.02gh	1.52±0.15abc	2.29±0.09ab	29.88±0.63cde
	40	6.33±0.33	32.67±1.84	0.96±0.05	65.50±0.84	2.96±0.20	3.92±0.09	33.28±1.11
H31	0	3.83±0.31bcd	31.00±0.65ab	0.59±0.04a	52.83±0.74defgh	1.05±0.10abc	1.64±0.09ab	26.55±0.99abcd
	40	6.50±0.22	30.83±0.92	0.93±0.06	64.67±1.07	2.76±0.13	3.69±0.09	32.67±1.00
H32	0	4.00±0.37de	32.83±1.27ab	0.83±0.15ab	52.33±1.01bcdefg	1.35±0.14abc	2.18±0.09ab	24.22±0.49ab
	40	8.00±0.45	31.08±1.31	1.38±0.06	60.33±0.88	3.54±0.08	4.91±1.09	31.28±0.70
H33	0	4.50±0.34de	28.33±0.95a	0.65±0.08ab	51.00±1.47abcde	1.00±0.12abc	1.65±0.09ab	26.72±0.96abcde
	40	7.83±0.48	29.25±0.82	0.99±0.08	58.17±2.11	3.02±0.26	4.00±0.09	33.08±1.02
H34	0	4.00±0.26bcde	29.42±1.19ab	0.67±0.04ab	54.08±1.37efgh	1.31±0.15abc	1.99±0.09ab	24.97±0.60ab
	40	7.33±0.42	31.08±1.54	1.22±0.04	65.42±0.60	3.31±0.23	4.52±0.09	30.53±0.77

H35	0	3.17±0.31ab	28.75±1.41a	0.53±0.05a	52.42±1.64cdefgh	1.19±0.11abc	1.72±1.09ab	28.25±1.09bcde
	40	5.00±0.26	28.83±0.65	1.01±0.19	63.50±1.13	2.68±0.42	3.69±0.09	33.97±0.67
H36	0	3.17±0.17abc	33.00±2.16ab	0.50±0.02a	54.92±0.85h	1.09±0.07ab	1.59±0.09ab	25.87±0.73abcd
	40	5.17±0.48	30.83±1.02	0.77±0.09	70.17±1.72	2.45±0.27	3.22±0.09	33.17±1.04
H37	0	3.33±0.21bcde	30.25±2.03ab	0.75±0.07ab	50.50±1.07bcdefg	1.18±0.08abc	1.94±0.09ab	24.82±0.75ab
	40	7.83±0.40	30.83±1.66	1.24±0.05	62.67±0.68	3.62±0.15	4.85±0.09	30.72±0.45
H39	0	3.80±0.37bcde	28.00±0.29a	0.77±0.10ab	51.25±0.99abcde	1.40±0.33abc	2.17±0.09ab	26.13±0.79abc
	40	7.50±0.62	28.67±1.23	1.05±0.13	57.67±1.54	3.02±0.23	3.39±0.09	32.02±0.49
I1	0	4.17±0.40bcde	35.17±1.12ab	0.63±0.03a	50.08±0.57abcdefg	1.32±0.07abc	1.95±0.09ab	28.43±0.92cde
	40	7.00±0.26	29.50±1.33	0.73±0.14	59.83±1.44	2.58±0.26	3.30±0.09	34.95±0.66
I2	0	3.50±0.22abcd	32.67±0.91b	0.63±0.10a	50.50±1.21abcd	1.13±0.14abc	1.76±0.09ab	27.60±0.54abc
	40	6.50±0.34	36.67±1.84	0.82±0.01	58.17±1.31	2.84±0.16	3.66±0.09	30.85±0.40
I4	0	3.50±0.22abcd	28.33±2.70ab	0.71±0.10a	53.42±1.70bcdefgh	1.36±0.21abc	2.07±0.09ab	31.13±0.42de
	40	5.83±0.48	31.00±1.70	0.87±0.11	61.50±0.75	2.26±0.26	3.13±1.09	34.50±1.21
I7	0	3.67±0.33bcde	32.42±0.92ab	0.52±0.06a	50.58±1.78abcdefg	1.06±0.13abc	1.57±0.09ab	28.22±0.64abcde
	40	7.00±0.52	29.58±1.39	0.85±0.08	60.92±2.15	2.60±0.19	3.45±0.09	33.13±1.06
I12	0	4.00±0.37bcde	26.92±0.86a	0.87±0.21ab	55.83±2.10gh	1.18±0.14abc	2.06±1.09ab	27.30±0.92cde
	40	7.33±0.76	28.92±1.14	1.08±0.16	64.42±2.96	3.19±0.45	4.28±0.09	36.33±3.58
I40	0	3.33±0.21bcde	31.08±1.07ab	0.59±0.06a	51.50±1.22bcdefgh	1.09±0.12abc	1.68±0.09ab	26.88±0.70abcde
	40	7.17±0.48	31.50±1.72	0.82±0.10	63.42±0.65	2.72±0.24	3.53±0.09	32.65±0.73
I41	0	3.17±0.31abcd	32.58±1.55ab	0.50±0.11a	47.00±1.63ab	0.95±0.21ab	1.46±1.09ab	26.23±0.37abcd
	40	6.50±0.34	29.58±1.23	0.82±0.09	57.67±1.31	2.53±0.15	3.35±0.09	32.95±0.63
I42	0	3.67±0.33bcde	30.58±1.00ab	0.59±0.02a	47.33±0.84a	0.99±0.09a	1.58±0.09a	27.12±0.64abcde
	40	6.67±0.92	30.00±2.58	0.83±0.17	54.08±1.99	2.23±0.39	3.06±1.09	32.40±1.04
I43	0	3.67±0.33bcde	30.33±0.83ab	0.66±0.17c	57.17±0.33h	1.34±0.11bc	2.00±0.09c	26.80±0.60a
	40	5.50±0.50	30.00±1.00	3.97±0.87	65.75±0.75	3.38±0.50	7.35±0.09	28.03±1.08
I44	0	2.83±0.17abcd	30.00±2.02ab	0.47±0.06ab	51.58±1.89cdefgh	0.89±0.15abc	1.36±0.09ab	30.32±0.98e
	40	6.33±0.33	31.92±2.04	1.12±0.14	64.00±1.45	3.03±0.14	4.16±1.09	36.23±1.12
I45	0	2.67±0.21a	31.92±1.51ab	0.49±0.06a	66.75±1.72i	1.18±0.11abc	1.67±0.09ab	29.03±0.95abcd
	40	4.00±0.45	30.83±0.71	0.88±0.22	78.17±1.34	2.79±0.22	3.67±0.09	35.58±1.00
I46	0	3.00±0.00bcd	30.58±0.08ab	0.53±0.04b	50.75±0.25cdefgh	0.67±0.02abc	1.21±0.09ab	29.47±0.86abcde
	40	7.17±0.17	27.67±0.67	2.05±0.15	65.25±1.25	3.61±0.33	5.66±0.09	30.47±0.64
F-Accession (A) [2]		4.897***	1.844***	7.587***	15.843***	2.352***	3.604***	5.327***
F-Nitrogen (N) [2]		918.770***	0.023	270.439***	871.242***	1087.228***	849.786***	465.505***
F-A×N [2]		2.508***	1.384	6.754***	1.228	1.930***	3.040***	1.743**
DF Error		313	314	313	314	313	315	314

1: Numbers are means ± standard errors ($N = 6$), lowercase letters indicate homogenous genotype groups based on Tukey LSD tests ($P > 0.05$). 2: F-values, accession DF = 31, nitrogen DF = 1, A×N DF = 31; ** = $P \leq 0.01$ and *** = $P \leq 0.001$.

Table S3. Results from greenhouse oviposition (performance) experiment with yellow stemborer.

Genotypes[1]	Number of Egg Masses Plant^{-1} [2]	Egg Mass Length (mm) [2]	Average Number of Eggs per Egg mass [2]	Number of Eggs Plant^{-1} [2]	Shoot Biomass (Dry g) [2]
H3	1.83±1.28	3.77±0.22	40.33±7.77	52.33±28.42	0.13±0.01ab
H14	1.17±0.65	3.85±0.05	33.17±3.74	38.83±21.29	0.14±0.01ab
H15	1.50±0.67	4.23±0.60	46.19±11.20	75.00±37.15	0.13±0.01ab
H16	2.00±0.37	3.65±0.49	30.03±7.99	62.33±18.16	0.10±0.00a
H17	1.67±0.56	3.81±0.57	44.25±9.20	87.50±38.41	0.14±0.01ab
H19	1.33±0.42	4.83±0.52	54.67±13.46	66.83±21.97	0.16±0.02b
H31	2.50±0.99	3.14±0.52	32.24±11.09	105.17±58.49	0.15±0.02b
H32	1.67±0.80	2.90±0.28	20.67±4.53	37.50±21.92	0.16±0.01b
H33	3.00±1.24	4.12±0.36	35.85±2.65	112.33±45.94	0.13±0.02ab
H34	3.50±1.06	4.39±0.27	44.53±5.31	156.00±52.27	0.12±0.01ab
H36	1.17±0.48	3.53±0.11	31.92±3.15	34.50±12.56	0.13±0.00ab
H37	1.67±0.42	3.80±0.51	42.83±9.28	95.40±28.17	0.16±0.01b
H39	4.83±1.11	4.07±0.60	42.55±7.72	180.83±37.56	0.13±0.00ab
I2	2.67±1.93	4.41±0.11	58.00±4.58	143.00±95.85	0.14±0.01ab
I4	2.33±0.92	4.10±0.29	49.68±6.58	121.00±48.88	0.13±0.01ab
I7	2.33±0.80	4.22±0.48	43.17±8.03	122.83±52.88	0.14±0.01ab
I12	1.83±0.54	4.01±0.48	46.30±9.59	79.33±25.96	0.11±0.00ab
I40	2.33±0.95	3.73±0.38	36.76±6.79	90.67±37.86	0.11±0.01ab
I42	1.50±0.56	2.95±0.42	24.95±7.95	53.83±36.42	0.11±0.01ab
I43	2.33±0.42	3.44±0.57	38.15±12.08	101.67±36.76	0.15±0.01b
I45	1.17±0.48	5.30±0.64	77.13±15.15	72.33±23.32	0.11±0.01ab
I46	1.17±0.48	3.18±0.32	26.25±3.36	32.33±14.38	0.11±0.00ab
F-Accession[3]	1.041	1.061	1.050	1.075	2.881***
DF Error	110	80	109	78	109

1: Plants of H1, H5, H9, H20, H30, H35, I1, I41 and I44 were unavailable for the experiment. 2: Numbers are means ± standard errors (N = 6), lowercase letters indicate homogenous genotype groups based on Tukey LSD tests (P > 0.05). 3: F-values, accession DF = 21, *** = P ≤ 0.001.

Table S4. Results from stemborer preference (antixenosis) and performance (antibiosis) experiments conducted in a screenhouse environment at the International Rice Research Institute.

Genotypes	Number of Tillers[1]	Plant Biomass (Dry g)[1]	Oviposition (Preference)[1]		Performance[1]					
			Number of Egg Masses Plant^{-1}	Number of Emerged Larvae Plant^{-1}	Pupal Weight (Dry mg)		Number of Individuals Plant^{-1}	Development to Adults (Proportion)	Total Stemborer Biomass (Dry mg Plant^{-1})	Dead Heart (Proportion of Tillers that were Dead)
					Female	Male				
H1	32.50±1.82cde	55.34±6.34b	0.50±0.34	17.67±13.17ab	12.00	11.00±0.53ab	5.17±1.19	0.17±0.06ab	24.73±5.56ab	18.59±5.06abc
H3	22.50±1.06abcde	34.46±7.24ab	1.33±0.42	59.33±20.79ab	20.06±2.52	9.34±0.38ab	9.00±1.39	0.63±0.09b	90.92±15.07b	34.08±5.68abcd
H5	34.83±3.15e	43.36±7.12ab	0.17±0.17	7.00±7.00ab	22.95±3.90	10.66±0.49ab	8.50±2.42	0.36±0.11ab	44.01±5.46ab	34.13±5.71abcd
H9	33.17±2.44cde	29.55±0.28ab	2.00±0.73	89.67±31.23ab	22.88±3.37	9.30±0.47b	9.17±2.57	0.23±0.05ab	49.57±10.10ab	48.65±7.95cd
H14	33.33±2.79de	28.23±4.44ab	1.17±0.54	79.67±36.52ab	23.80±3.45	7.43±0.63ab	8.50±1.80	0.18±0.05ab	41.92±9.20ab	37.91±4.51abcd
H15	33.50±3.25de	44.56±8.18ab	1.17±0.48	41.50±16.71ab	17.01±2.91	8.31±0.76ab	11.33±1.31	0.30±0.09ab	56.45±14.31ab	45.97±7.57bcd
H16	25.00±1.37bcde	35.62±8.17ab	0.50±0.22	26.00±17.14ab	15.20±4.30	9.06±0.45ab	9.17±2.52	0.24±0.09ab	40.21±12.16ab	35.06±6.19abcd
H17	31.83±4.02bcde	29.99±3.99ab	1.17±0.31	35.50±14.30ab	14.33±3.15	9.17±0.16ab	10.67±2.42	0.26±0.07ab	44.20±8.40ab	38.70±6.32abcd
H18	20.50±1.61abcde	47.20±5.85ab	0.67±0.49	27.83±25.70ab	16.53±2.42	10.17±0.85ab	7.50±1.18	0.25±0.06ab	41.67±6.59ab	27.25±5.46ab
H19	36.17±3.11e	47.32±3.25ab	1.83±0.60	118.83±38.53b	20.74±3.07	9.85±0.72ab	9.17±1.70	0.53±0.07ab	75.31±15.09ab	36.65±4.17bcd
H20	28.67±3.60bcde	50.86±16.99ab	0.50±0.22	20.17±10.34ab	19.54±2.53	9.29±0.61ab	11.50±2.01	0.40±0.05ab	67.19±8.34ab	47.05±7.14abcd
H30	24.67±2.82bcde	41.50±5.30ab	1.00±0.68	60.50±43.54ab	16.62±2.60	9.66±0.35ab	10.00±1.46	0.36±0.10ab	63.51±17.01ab	38.27±10.25abcd
H31	34.67±3.57de	33.40±5.55ab	1.17±0.40	45.83±14.97ab	23.05±2.32	8.73±0.83ab	9.17±2.18	0.52±0.11ab	74.07±12.78ab	50.92±6.22d
H32	33.00±3.84cde	28.45±3.19ab	0.50±0.34	16.50±10.78ab	NA	8.34±0.99ab	7.50±1.77	0.21±0.09ab	29.95±6.77ab	36.21±8.21abcd
H33	27.83±3.68bcde	40.61±9.36ab	0.33±0.33	12.17±12.17ab	20.35±3.95	8.82±0.33ab	8.67±1.43	0.45±0.11ab	83.77±29.97ab	45.76±9.85abcd
H34	27.17±2.97bcde	36.60±6.83ab	1.67±0.49	72.00±23.55ab	21.88±4.16	9.46±1.07ab	10.00±2.07	0.26±0.07ab	42.67±3.58ab	40.45±6.22abcd
H35	17.00±1.46abc	26.23±5.76ab	1.33±0.42	91.83±36.69ab	21.89±2.30	9.73±0.55ab	8.00±1.26	0.39±0.06ab	66.59±12.15ab	46.86±7.98abcd
H36	15.67±1.58ab	38.28±9.24ab	0.67±0.33	43.83±21.41ab	13.01±1.77	8.68±0.63ab	8.50±0.92	0.31±0.10ab	42.82±8.06ab	49.68±4.32abcd
H37	30.17±4.09bcde	27.77±3.62ab	1.17±0.54	55.67±26.14ab	20.17±2.23	9.58±0.99ab	9.17±2.04	0.39±0.10ab	64.97±14.90ab	37.44±6.70abcd
H39	34.33±4.01de	40.74±3.22ab	1.50±0.34	63.17±33.03ab	17.89±2.57	11.20±0.51ab	9.83±1.30	0.30±0.05ab	60.34±8.92ab	33.15±4.75abcd
I1	35.67±1.80e	29.78±7.85ab	1.33±0.56	54.00±18.28ab	19.20±0.90	8.60±1.67ab	5.83±1.33	0.31±0.16ab	25.42±5.31ab	31.59±6.42abcd
I2	35.67±3.58e	44.36±12.59ab	1.00±0.37	22.33±7.64ab	19.33±1.97	9.60±0.50ab	9.00±1.26	0.39±0.06ab	69.40±10.40ab	47.88±9.81bcd
I4	17.00±1.00abc	20.01±2.26ab	1.00±0.26	24.33±7.09ab	20.80±0.70	10.27±0.99ab	4.83±1.76	0.19±0.08ab	33.62±8.05ab	39.91±6.39abcd
I7	27.67±3.07bcde	25.71±6.57ab	0.67±0.33	42.17±19.94b	20.02±4.87	8.82±0.99ab	10.83±2.46	0.41±0.05ab	80.57±20.87ab	46.40±3.57abcd
I12	30.50±2.72bcde	44.47±10.24ab	0.50±0.22	7.50±4.75ab	23.19±2.41	8.94±0.42ab	9.17±2.27	0.38±0.08ab	69.98±21.20ab	43.29±6.96abcd
I40	30.00±2.86bcde	32.21±3.45ab	1.33±0.49	34.83±17.49ab	13.50	8.43±0.95a	6.17±2.07	0.28±0.11ab	25.59±10.60ab	48.77±4.63bcd
I41	26.67±1.09bcde	31.93±5.53ab	1.17±0.17	47.17±15.94ab	20.30±5.00	8.48±0.64ab	9.00±1.53	0.24±0.05ab	39.12±8.80ab	45.87±6.92abcd
I42	29.67±3.31bcde	31.95±6.89ab	0.50±0.22	16.17±8.21ab	14.10±2.80	8.81±1.16ab	6.00±1.51	0.22±0.10ab	35.90±10.51ab	35.02±7.31abcd
I43	24.50±3.72bcde	16.95±1.44ab	0.67±0.49	25.50±22.45ab	17.65±3.25	9.12±0.89ab	8.83±1.47	0.45±0.05ab	66.93±16.45ab	50.13±8.24abcd
I44	27.00±5.74bcde	15.97±5.85a	1.17±0.40	76.83±38.88ab	24.29±0.71	9.68±0.33ab	6.17±2.06	0.39±0.16ab	60.84±20.02ab	35.75±4.79abcd

I45	8.00±0.86a	19.72±3.98ab	1.17±0.54	66.33±31.29ab		11.00±0.70ab	3.83±0.83	0.14±0.07a	18.22±3.84a	38.74±5.54a
I46	18.50±2.54abcd	24.96±8.09ab	0.00±0.00	0.00±0.00a	16.92±3.06	8.27±1.15ab	8.50±2.51	0.39±0.08ab	62.57±19.87ab	43.88±5.08abcd
DF	31.00	31.00	31	31	31.00		31	31	31.00	31.00
DF	160.00	64.00	160	160	196.00		159	154	159.00	160.00
F-Accession (A) [2]	5.366***	2.017**	1.410ns	1.586*	1.540*		1.002	1.817*	2.169***	2.911***
F-Nitrogen (N) [2]					186.674***					
F-A×N [2]					1.647*					
F-Tillers [2]							8.980***	6.782*	6.155*	
DF Error	160	160	160	160	196		159	154	159	160

1: Numbers are means ± standard errors (N = 6), lowercase letters indicate homogenous genotype groups based on Tukey LSD tests (P > 0.05). 2: F-values, accession DF = 31, nitrogen DF = 1, tillers (covariate) DF = 1; A×N DF = 31; * = P ≤ 0.05, ** = P ≤ 0.01, and *** = P ≤ 0.001.

Table S5. Results of adult settling and oviposition (preference) experiments with brown planthopper (BPH) and whitebacked planthopper (WBPH) in greenhouse cages.

Genotypes	BPH[2]				WBPH[2]			
	Adults Settled after 72 Hours	Number of Egg Masses Plant^{-1}	Number of Eggs Plant^{-1}	Shoot Biomass (Dry g)	Adults Settled after 72 Hours[2]	Number of Egg Masses Plant^{-1}	Number of Eggs Plant^{-1}	Shoot Biomass (Dry g)
H1	0.33±0.21a	10.50±3.79ab	49.17±18.57ab	0.08±0.01abcdef	0.67±0.67abc	4.33±2.33a	20.50±13.99a	0.09±0.01abc
H3	0.33±0.33a	25.83±7.59ab	135.50±40.99ab	0.11±0.01defg	0.67±0.49abc	11.00±3.17ab	66.50±16.65ab	0.11±0.01abcdef
H5	1.33±0.84ab	17.20±6.36ab	87.20±37.28ab	0.06±0.01abc				
H9	3.33±1.67ab	28.50±11.47ab	167.67±66.24ab	0.08±0.01abcdef	0.33±0.21abc	7.67±3.30ab	43.17±18.95ab	0.08±0.02abc
H14	1.17±0.48ab	26.00±4.42ab	145.00±23.78ab	0.12±0.01fg	0.67±0.42abc	4.33±1.45ab	22.50±8.78a	0.11±0.02abcde
H15	1.00±0.37ab	15.00±5.39ab	84.00±34.17ab	0.06±0.00abcd	0.00±0.00a	3.50±2.54a	21.00±14.01a	0.08±0.01abc
H16	0.17±0.17a	8.17±4.42ab	47.00±30.62ab	0.07±0.00abcde	0.00±0.00a	5.67±0.99ab	35.67±8.07ab	0.08±0.01abc
H17	0.33±0.21a	14.50±6.71ab	72.33±31.76ab	0.10±0.00bcdefg	0.00±0.00a	3.00±1.03a	16.67±5.25a	0.09±0.01abc
H19	2.00±0.58ab	22.00±7.32ab	147.50±56.65ab	0.11±0.02efg				
H20	2.00±0.93ab	29.83±10.33ab	159.33±44.25ab	0.12±0.01g	2.17±0.75abcd	11.83±5.13ab	67.17±26.89ab	0.15±0.01ef
H30	5.33±0.84b	44.17±8.74b	245.17±33.86b	0.11±0.01efg	2.00±0.45bcd	13.00±3.85ab	66.00±19.67ab	0.09±0.01abcd
H31	3.17±1.72ab	22.83±8.96ab	133.50±53.58ab	0.10±0.00cdefg	1.00±0.52abcd	8.17±2.30ab	63.67±20.01ab	0.13±0.01abcdef
H32	2.33±0.71ab	30.50±11.09ab	186.17±44.71ab	0.10±0.01abcdefg	0.67±0.33abcd	5.00±1.83ab	32.83±13.52ab	0.14±0.01cdef
H33	0.67±0.49ab	14.00±4.95ab	82.83±24.63ab	0.11±0.02efg	0.17±0.17ab	2.33±0.80a	12.17±3.37a	0.11±0.01abcde
H34	0.83±0.31ab	9.00±2.21ab	65.50±18.03ab	0.07±0.01abcde	1.33±0.88abcd	8.00±2.34ab	47.83±14.36ab	0.11±0.01abcdef
H36	1.33±0.42ab	16.83±4.11ab	122.50±34.03ab	0.09±0.00abcdefg	0.50±0.34abc	8.83±3.83ab	61.83±28.32ab	0.15±0.01def
H37	0.33±0.21a	7.00±2.80ab	49.17±21.35ab	0.10±0.01bcdefg	0.33±0.21abc	4.83±1.58ab	27.33±10.10ab	0.16±0.01f
H39	0.33±0.21a	24.00±16.22ab	131.67±86.58ab	0.09±0.00abcdefg	0.17±0.17ab	5.00±1.90ab	24.83±9.73ab	0.13±0.01bcdef
I1	1.60±0.60ab	26.83±9.42ab	165.83±50.14ab	0.06±0.01abc	0.50±0.29abcd	5.50±2.61ab	43.00±20.28ab	0.08±0.00ab
I2	0.50±0.22ab	6.00±1.59ab	41.17±12.45ab	0.06±0.00abc	5.50±1.45d	24.17±2.04b	134.67±9.53b	0.09±0.01abc
I4	1.33±0.80ab	13.67±8.38ab	87.17±46.62ab	0.08±0.01abcdef	1.00±0.82abcd	8.33±4.67ab	45.00±27.30ab	0.11±0.01abcdef
I7	0.50±0.22ab	3.50±1.50a	23.67±10.78a	0.06±0.01abc	0.83±0.54abcd	10.33±2.76ab	63.17±15.57ab	0.14±0.01cdef
I12	0.83±0.65ab	7.33±2.97ab	45.33±20.63ab	0.05±0.00a	0.33±0.33abc	5.50±0.67ab	20.67±4.21a	0.08±0.01abc
I40	2.67±0.99ab	16.83±8.50ab	116.83±58.75ab	0.06±0.00ab	2.17±0.95abcd	12.67±4.25ab	66.00±22.56ab	0.11±0.01abcde
I41	0.00±0.00a	5.33±1.93ab	35.00±12.73ab	0.08±0.01abcdef	0.67±0.21abcd	5.00±0.77ab	26.83±3.18ab	0.11±0.01abcde
I42	1.33±0.56ab	13.83±2.86ab	83.17±12.86ab	0.07±0.01abcde	3.67±0.84cd	18.33±6.41ab	108.83±33.79ab	0.09±0.01abcd
I43	1.17±0.48ab	12.33±3.37ab	92.67±23.71ab	0.09±0.00bcdefg	2.00±0.68abcd	13.67±3.91ab	68.00±18.62ab	0.08±0.00abc
I44	0.50±0.50a	6.17±2.77ab	38.33±17.82ab	0.06±0.00abc	1.33±0.88abcd	7.00±2.16ab	37.17±10.62ab	0.08±0.01abc
I45	2.67±0.56ab	21.67±7.45ab	138.83±46.08ab	0.07±0.00abcd	3.00±1.21abcd	18.17±6.81ab	99.83±37.85ab	0.08±0.00abc
I46	1.50±0.96ab	19.17±12.74ab	87.33±58.67ab	0.05±0.01a	0.83±0.40abcd	8.17±2.50ab	41.00±11.07ab	0.08±0.01a
F-Accession[3]	2.390***	1.942**	1.964***	6.950***	3.513***	2.153***	2.611***	6.133***
DF Error	149	149	149	149	137	137	137	137

1: Plants of H18, H35 were unavailable for the experiments with BPH and WBPH, plants of H5 and H19 were unavailable for experiments with WBPH. 2: Numbers are means ± standard errors (N = 6), lowercase letters indicate homogenous genotype groups based on Tukey LSD tests ($P > 0.05$). 3: F-values, accession DF = 29 for BPH and 27 for WBPH, ** = $P \leq 0.01$ and *** = $P \leq 0.005$ for data ranked within replicates.

Table S6. Performance (population build-up) of brown planthoppers (BPH) on 32 rice genotypes in a greenhouse experiment.

Genotypes	Applied Nitrogen (Kg ha^{-1})	Development to Adults (Proportion)[1]	Brachypterous Adults (Proportion)[1]	Adult Females (Proportion)[1]	Number of Planthoppers Plant^{-1} [1]	First Sample BPH Biomass (Dry mg)[1]	Second Sample BPH Biomass (Dry mg)[1]
H1	0	0.97±0.01	0.99±0.01bc	0.63±0.02ab	142.25±37.25	96.32±19.83b	114.89±27.84ab
	150	0.96±0.01	0.96±0.03	0.55±0.01	180.75±20.41	92.20±12.41	104.75±23.72
H3	0	0.98	1.00±0.00c	0.64±0.04ab	79.25±18.30	36.28±7.80ab	40.29±10.95ab
	150	0.93	1.00±0.00	0.60±0.03	263.50±95.37	99.18±29.32	135.56±26.85
H5	0	0.87±0.07	0.97±0.01bc	0.59±0.05ab	142.83±51.45	96.70±31.30b	141.08±31.94b
	150	0.70±0.30	0.98±0.01	0.67±0.02	153.00±140.21	117.43±29.80	127.63±29.38
H9	0	0.88±0.13	0.97±0.02abc	0.62±0.05ab	189.25±84.58	95.56±39.88b	78.46±35.35ab
	150	1.00±0.00	0.92±0.02	0.58±0.03	172.75±5.50	104.58±3.78	129.84±16.92
H14	0	0.96±0.02	0.95±0.02abc	0.54±0.02ab	193.50±52.94	67.56±25.79b	77.98±22.60ab
	150	0.98±0.01	0.99±0.01	0.56±0.01	150.75±34.83	95.38±13.63	117.58±16.72
H15	0	0.94±0.05	0.97±0.02bc	0.57±0.03ab	104.50±44.08	47.75±13.40b	68.21±13.48ab
	150	0.72±0.17	0.98±0.02	0.57±0.02	400.50±240.27	129.30±18.78	150.48±24.79
H16	0	0.94±0.02	0.99±0.00abc	0.51±0.04ab	268.00±118.76	63.49±10.49ab	97.28±21.93ab
	150	0.90±0.07	0.95±0.05	0.67±0.02	173.25±80.40	79.29±25.84	144.18±24.04
H17	0	0.89±0.04	0.98±0.02bc	0.54±0.04ab	113.75±33.22	48.37±9.51ab	82.93±23.82ab
	150	0.92±0.04	0.99±0.01	0.50±0.00	158.50±43.76	91.81±18.48	137.24±37.16
H18	0	0.98±0.02	0.98±0.02bc	0.63±0.02ab	99.25±44.18	49.84±33.53b	56.68±31.63ab
	150	0.97±0.01	0.97±0.00	0.58±0.02	270.67±17.37	135.41±18.63	155.63±22.40
H19	0	0.92±0.04	0.97±0.03abc	0.54±0.01ab	112.50±14.93	36.01±5.13ab	36.01±5.13ab
	150	0.98±0.01	0.84±0.10	0.61±0.04	175.50±4.94	90.67±29.82	114.00±51.34
H20	0	0.85±0.06	0.99±0.00bc	0.42±0.11ab	231.33±119.23	71.62±30.62b	72.67±30.54ab
	150	0.98±0.01	0.97±0.02	0.57±0.05	275.67±99.35	92.92±13.97	137.93±19.95
H30	0	0.68±0.16	0.93±0.04abc	0.44±0.12ab	155.50±101.01	75.90±29.01ab	85.68±25.69ab
	150	0.93±0.05	0.98±0.00	0.59±0.03	132.50±53.95	64.09±20.39	119.33±32.33
H31	0	0.95±0.03	0.99±0.01bc	0.54±0.02ab	253.75±107.04	101.75±20.46b	102.80±21.20ab
	150	0.68±0.16	0.98±0.01	0.61±0.04	350.75±117.39	104.07±18.66	131.65±26.55
H32	0	0.74±0.25	0.97±0.03bc	0.31±0.16ab	114.25±60.13	56.83±7.01ab	56.83±7.01ab
	150	0.61±0.35	1.00±0.00	0.76±0.01	281.50±129.71	63.42±13.70	77.95±11.02
H33	0	0.94±0.02	0.97±0.03abc	0.46±0.06ab	116.50±21.48	92.88±20.01b	97.38±20.39ab
	150	0.97±0.02	0.97±0.02	0.57±0.01	149.00±56.77	101.55±23.33	111.67±18.84
H34	0	1.00	0.94abc	0.63ab	121.00±59.76	65.86±31.94ab	83.51±24.70ab
	150	0.96±0.04	0.84±0.16	0.64±0.04	137.50±47.81	76.42±35.95	79.32±38.26
H35	0	0.96	1.00±0.00abc	0.61±0.04ab	111.50±46.52	32.00±10.31ab	57.19±16.67ab
	150	0.97±0.03	0.93±0.03	0.53±0.04	181.20±30.72	73.57±22.03	94.18±40.85
H36	0	0.84±0.16	0.97±0.03abc	0.48±0.02ab	184.75±53.16	88.75±28.88b	88.75±28.88ab
	150	0.97±0.01	0.90±0.09	0.54±0.11	214.25±49.03	127.41±22.74	151.82±41.56

H37	0	0.98±0.02	0.99±0.01bc	0.61±0.05ab	138.00±45.60	60.38±21.56ab	57.97±39.27ab
	150	0.91±0.09	0.98±0.02	0.58±0.03	201.67±83.30	96.25±24.51	87.57±23.53
H39	0	0.83±0.16	0.90±0.03abc	0.54±0.21ab	136.33±75.27	73.21±11.42b	79.09±10.74ab
	150	0.94±0.03	0.99±0.01	0.52±0.04	225.75±87.77	84.32±27.41	112.17±23.68
I1	0	0.89±0.04	0.93±0.02abc	0.61±0.13b	135.00±68.07	62.79±23.20ab	102.93±28.49ab
	150	0.95±0.02	0.99±0.00	0.68±0.07	85.33±42.72	87.71±17.30	137.16±21.40
I2	0	0.91±0.07	0.99±0.01abc	0.59±0.07ab	122.75±35.65	45.45±9.18ab	73.49±15.28ab
	150	0.88±0.10	0.94±0.06	0.53±0.04	156.25±62.39	114.37±38.56	151.82±42.38
I4	0	1.00±0.00	0.97±0.02bc	0.49±0.03ab	176.75±42.18	77.50±16.31b	94.96±10.94ab
	150	0.98±0.01	1.00±0.00	0.53±0.01	250.00±33.67	94.54±18.19	136.36±25.10
I7	0	0.84±0.14	0.75±0.14a	0.59±0.07ab	70.75±17.67	50.59±6.83b	86.37±8.63ab
	150	0.98±0.00	0.90±0.08	0.55±0.03	179.00±59.63	99.69±23.39	154.51±24.71
I12	0	0.80±0.17	1.00±0.00c	0.80±0.00b	150.25±93.02	81.65±28.52b	94.56±27.61b
	150	0.97±0.02	0.99±0.01	0.56±0.03	405.50±64.53	182.82±22.04	208.00±28.46
I40	0	1.00±0.00	0.98±0.00bc	0.60±0.09ab	77.00±2.89	40.55±7.27b	46.72±7.68ab
	150	0.97±0.02	0.98±0.01	0.54±0.02	365.50±22.84	118.72±24.81	146.55±30.28
I41	0	0.79±0.10	1.00±0.00c	0.64±0.06ab	119.67±103.20	63.50±19.86ab	71.00±25.91ab
	150	0.67±0.11	0.99±0.01	0.47±0.11	64.67±20.51	82.50±29.68	93.90±25.75
I42	0	0.90±0.10	0.90±0.06abc	0.61±0.03ab	20.00±5.00	15.97±3.76a	23.48±7.70a
	150	0.56±0.16	1.00±0.00	0.67±0.11	82.67±37.71	33.60±10.67	43.89±12.76
I43	0	0.99±0.01	0.77±0.23ab	0.53±0.07ab	251.67±212.92	68.71±25.53ab	68.71±25.53ab
	150	0.96±0.02	0.92±0.06	0.57±0.09	41.33±27.34	60.26±12.72	83.57±29.14
I44	0	0.53±0.33	0.94±0.05abc	0.52±0.03ab	331.00±205.01	76.97±18.18b	81.91±19.01ab
	150	0.96±0.04	1.00±0.00	0.64±0.11	245.25±71.70	138.38±48.59	182.41±46.35
I45	0	1.00±0.00	0.98±0.02abc	0.57±0.03ab	156.75±41.49	77.79±7.83b	77.79±7.83ab
	150	0.99±0.01	0.96±0.03	0.57±0.04	170.50±40.45	108.95±18.24	122.94±12.34
I46	0	0.45±0.08	0.99±0.00abc	0.50±0.02a	306.50±141.32	69.71±26.20ab	71.29±26.12ab
	150	0.98	0.86	0.26	219.33±74.90	70.37±19.94	76.27±17.04
F-Accession (A) [2]		1.505	1.811*	1.630*	0.95	2.376***	1.735*
F-Nitrogen (N) [2]		0.548	0.001	1.042	5.835*	32.879***	50.958***
F-A×N [2]		1.218	1.325	2.335	0.925	0.859	0.873
DF Error		102	145	136	195	251	251

1: Numbers are means ± standard errors (N = 6), lowercase letters indicate homogenous genotype groups based on Tukey LSD tests (P > 0.05). 3: F-values, accession DF = 31, Nitrogen DF = 1, A × N DF = 31, * = P ≤ 0.05 and *** = P ≤ 0.005

Table S7. Performance (population build-up) of whitebacked planthoppers (WBPH) on 32 rice genotypes in a greenhouse experiment.

Genotypes	Applied Nitrogen (Kg ha^{-1})	Development to Adults (Proportion)[1]	Brachypterous Adults (Proportion)[1]	Adult Females (Proportion)[1]	Number of Planthoppers Plant^{-1} [1]	First Sample WBPH Biomass (Dry mg)[1]	Second Sample WBPH Biomass (Dry mg)[1]
H1	0	0.96±0.04	0.71±0.13	0.74±0.07	17.50±2.60a	16.58±11.51abc	25.52±16.59abc
	150	0.39±0.28	0.90±0.04	0.87±0.01	169.75±69.80	19.82±2.36	41.88±10.32
H3	0	0.21±0.20	0.95±0.05	0.95±0.05	203.33±135.57ab	14.17±2.69abc	26.65±7.76abc
	150	0.04±0.01	0.81±0.18	0.96±0.03	812.67±202.76	23.81±3.54	59.45±20.73
H5	0	0.86±0.14	0.80±0.20	0.97±0.03	12.67±11.17a	4.47±1.12abc	7.04±2.06abc
	150	0.44±0.28	0.83±0.13	0.83±0.13	108.33±65.38	12.99±4.12	37.53±13.77
H9	0	0.51±0.32	0.54±0.21	0.73±0.06	34.50±18.50a	8.54±2.92abc	13.81±4.43abc
	150	0.43±0.40	0.65±0.27	0.84±0.08	264.00±234.00	14.22±1.78	18.89±2.60
H14	0	0.64±0.27	0.48±0.15	0.72±0.08	101.00±36.18a	11.84±7.02abc	14.87±7.92abc
	150	0.75±0.25	0.62±0.14	0.85±0.02	92.00±8.78	19.49±7.28	39.33±19.44
H15	0	0.78±0.12	0.47±0.31	0.64±0.14	14.50±5.50a	7.92±3.57abc	12.66±5.08abc
	150	0.61±0.22	0.60±0.22	0.84±0.08	118.50±57.02	39.13±19.33	73.33±29.81
H16	0	0.98±0.02	0.77±0.09	0.75±0.16	25.67±10.65a	9.00±1.09abc	13.82±2.85abc
	150	0.38±0.31	0.48±0.24	0.67±0.09	345.33±156.17	21.38±5.26	45.50±11.48
H17	0	1.00	1.00	1.00	6.25±2.10a	3.33±0.97a	3.98±1.51a
	150	1.00±0.00	1.00±0.00	0.83±0.17	8.00±5.02	3.11±1.45	4.56±2.72
H18	0	0.65±0.30	0.84±0.13	0.81±0.16	80.50±19.60a	16.88±4.39abc	24.89±7.40abc
	150	0.45±0.27	0.71±0.08	0.71±0.07	200.00±43.69	26.44±4.76	56.99±13.39
H19	0	0.38±0.31	0.63±0.19	0.88±0.07	284.67±218.46ab	13.18±3.35abc	20.17±5.01abc
	150	0.17	0.77	0.93	290.00±110.00	34.99±6.22	47.39±6.22
H20	0	0.41±0.24	0.85±0.11	0.97±0.03	75.50±32.32a	7.85±0.95abc	11.46±1.58abc
	150	0.62±0.23	0.53±0.27	0.82±0.05	201.67±85.71	36.73±4.58	65.77±12.99
H30	0	0.94	0.81	0.79	53.00±3.00ab	12.03±2.11abc	17.96±2.38abc
	150	0.51±0.49	0.80±0.20	0.90±0.10	458.00±455.00	30.03±4.69	46.66±7.46
H31	0	0.67±0.33	0.63±0.31	0.96±0.03	60.00±33.08ab	8.89±1.54abc	12.39±2.07abc
	150	0.05±0.04	0.97±0.03	0.97±0.03	746.67±529.50	22.43±5.76	48.45±20.55
H32	0	0.92±0.08	0.67±0.09	0.87±0.02	43.67±16.60ab	10.24±4.26abc	14.67±8.05abc
	150	0.03±0.00	0.74±0.24	0.74±0.24	875.00±809.00	26.21±5.02	36.19±10.77
H33	0	0.70±0.30	0.85±0.12	0.87±0.13	25.33±7.62a	8.90±1.72abc	16.74±5.20abc
	150	0.57±0.43	0.66±0.26	0.96±0.04	132.00±43.00	16.83±7.14	32.65±18.55
H34	0	0.37±0.32	0.79±0.09	0.83±0.05	430.00±212.47ab	19.23±3.03abc	37.82±8.06bc
	150	0.03±0.01	0.58±0.25	0.78±0.07	1203.33±393.93	34.07±9.14	57.77±20.32
H35	0	0.77±0.16	0.56±0.27	0.82±0.05	111.00±61.72ab	21.88±7.06abc	33.18±12.72abc
	150	0.06±0.04	0.84±0.16	0.84±0.16	299.33±94.44	25.63±6.94	52.40±10.86

H36	0	0.44±0.28	0.85±0.04	0.94±0.03	117.33±49.67ab	21.99±4.28bc	37.20±8.57c
	150	0.31±0.14	0.65±0.26	0.85±0.06	382.67±210.48	34.90±3.45	61.89±13.41
H37	0	0.38±0.26	0.89±0.06	0.97±0.03	254.00±218.31ab	12.61±2.58abc	21.12±5.73abc
	150	0.03±0.01	0.84±0.08	0.90±0.02	1458.00±314.00	35.32±6.74	49.12±11.96
H39	0	0.75±0.19	0.76±0.24	0.93±0.07	22.33±4.37a	5.11±2.26abc	6.57±2.16abc
	150	0.39±0.31	0.93±0.04	0.96±0.04	307.33±204.09	17.53±4.67	36.69±13.02
I1	0	0.74±0.26	0.59±0.30	0.82±0.09	117.67±108.67a	13.26±4.63abc	22.68±5.30abc
	150	0.41±0.30	0.73±0.20	0.90±0.05	161.00±104.62	33.69±13.58	54.05±9.88
I2	0	0.96±0.03	0.81±0.06	0.84±0.09	56.50±40.50a	14.67±7.24abc	26.26±9.94abc
	150	0.67±0.26	0.60±0.20	0.82±0.08	187.67±164.18	26.40±7.07	48.99±10.35
I4	0	0.66±0.30	0.85±0.15	0.85±0.15	79.67±64.83a	14.13±2.77abc	31.96±4.43abc
	150	0.42±0.29	0.60±0.30	0.93±0.04	249.67±134.52	31.45±6.36	57.37±8.35
I7	0	0.46±0.23	0.33±0.12	0.63±0.05	176.67±103.91ab	18.38±4.04abc	34.87±10.86abc
	150	0.34±0.33	0.70±0.03	0.76±0.05	378.67±272.55	21.70±5.37	32.49±6.99
I12	0	0.97±0.03	0.35±0.15	0.71±0.11	18.67±11.61a	6.51±2.04abc	7.05±1.89abc
	150	0.95±0.05	0.69±0.19	0.94±0.06	7.00±3.00	25.99±15.84	32.94±16.98
I40	0	0.50±0.50	0.47±0.47	0.50±0.50	204.00±187.00ab	13.66±4.30abc	17.79±6.45abc
	150	0.07±0.03	0.83±0.11	0.83±0.11	381.00±164.93	32.13±8.61	53.29±12.20
I41	0	0.52±0.29	0.90±0.10	0.90±0.10	3.67±2.67a	0.85±0.43a	1.40±0.87ab
	150	0.44±0.20	0.46±0.24	0.73±0.08	74.33±56.41	4.69±1.31	10.13±4.26
I42	0	0.54±0.46	0.20±0.20	0.65±0.15	16.00±11.00a	1.34±0.27ab	1.34±0.27ab
	150	0.98±0.02	0.63±0.23	0.81±0.05	23.50±1.50	8.44±2.09	10.80±2.77
I43	0	0.55±0.45	0.72±0.09	0.73±0.11	301.00±248.00ab	22.28±3.87abc	30.13±5.48abc
	150	0.15±0.06	0.69±0.22	0.83±0.09	809.33±670.29	23.84±8.70	42.26±19.41
I44	0	0.05±0.05	0.77±0.17	0.87±0.07	497.00±313.00b	17.19±2.43bc	35.40±3.65c
	150	0.21±0.20	0.62±0.12	0.81±0.09	1456.00±745.16	38.32±5.66	64.97±7.75
I45	0	0.75±0.23	0.51±0.11	0.62±0.00	217.00±131.79ab	22.09±6.81c	35.11±11.93bc
	150	0.27±0.25	0.78±0.07	0.78±0.07	1000.00±525.73	39.96±13.09	59.15±16.92
I46	0	0.66±0.23	0.47±0.09	0.58±0.02	93.67±13.68ab	14.90±2.89abc	27.39±6.90abc
	150	0.67±0.32	0.59±0.20	0.73±0.12	523.00±445.51	22.13±3.76	35.44±8.20
F-Accession (A) [2]		1.331	0.794	1.662*	2.459***	2.497***	2.408***
F-Nitrogen (N) [2]		13.959***	0.475	1.926	30.151***	66.397***	75.540***
F-A×N [2]		0.589	0.774	0.851	0.969	0.768	0.73
DF Error		108	108	110	122	288	293

1: Numbers are means ± standard errors (N = 6), lowercase letters indicate homogenous genotype groups based on Tukey LSD tests (P > 0.05).2: F-values, accession DF = 31, Nitrogen DF = 1, A × N DF = 31, * = P ≤ 0.05 and *** = P ≤ 0.005

Table S8. Results from monitoring of field plots at 35 DAS. The numbers of whitebacked planthoppers (WBPH), brown planthopper (BPH) and *Nephotettix* spp. (virus vectors) were counted and the number of tillers per plant infested with yellow stemborer (YSB) and striped stemborer (SSB)

Genotypes	Number of WBPH Plant^{-1} [1]	Number of BPH Plant^{-1} [1]	Number of Virus Vectors (*Nephotettix* spp.) [1]	Total Number of Leafhoppers and Planthoppers Plant^{-1} [1]	Number of YSB Sample^{-1} [1,2]	Number of SSB Sample^{-1} [1,2]	Percentage Deadheart Plot^{-1} [1]
H1	4.11±1.48ab	0.17±0.17	0.44±0.44	4.28±1.58ab	0.67±0.33	5.67±2.60ab	2.04±1.19ab
H3	2.83±0.84ab	0.11±0.11	0.06±0.06	2.94±0.95ab	2.00±1.15	16.67±8.69ab	7.17±4.47ab
H5	2.50±0.48ab	0.28±0.20	0.22±0.06	2.78±0.64ab	0.00±0.00	3.33±1.86ab	0.69±0.35a
H9	3.44±0.64ab	0.28±0.06	0.33±0.25	3.72±0.65ab	1.00±0.58	4.33±2.33ab	1.50±1.12ab
H14	2.50±0.42ab	0.11±0.11	0.28±0.20	2.61±0.47ab	0.67±0.33	8.00±5.13ab	3.23±2.35ab
H15	3.39±0.44ab	0.06±0.06	0.06±0.06	3.44±0.39ab	0.00±0.00	1.33±0.33ab	0.15±0.01a
H16	3.28±0.62ab	0.06±0.06	0.11±0.06	3.33±0.67ab	0.33±0.33	8.00±7.02ab	3.43±3.32ab
H17	1.33±0.44a	0.00±0.00	0.00±0.00	1.33±0.44a	0.67±0.67	2.67±0.67ab	1.14±0.63ab
H18	3.17±0.76ab	0.11±0.11	0.11±0.11	3.28±0.81ab	0.33±0.33	8.00±3.46ab	4.23±3.20ab
H19	3.67±1.42ab	0.11±0.06	0.11±0.06	3.78±1.43ab	0.00±0.00	5.00±2.08ab	1.10±0.63ab
H20	5.44±1.56b	0.11±0.11	0.28±0.15	5.56±1.49b	0.00±0.00	12.00±3.06ab	2.85±1.27ab
H30	5.39±1.35b	0.06±0.06	0.22±0.15	5.44±1.40b	0.00±0.00	14.00±10.02ab	3.82±2.86ab
H31	2.78±0.91ab	0.06±0.06	0.00±0.00	2.83±0.96ab	0.00±0.00	4.67±2.33ab	1.15±0.70ab
H32	2.72±0.55ab	0.06±0.06	0.22±0.15	2.78±0.56ab	0.00±0.00	2.00±1.53ab	0.49±0.43a
H33	2.44±0.29ab	0.11±0.06	0.17±0.10	2.56±0.24ab	0.00±0.00	0.67±0.67a	0.19±0.19a
H34	3.50±1.18ab	0.11±0.06	0.28±0.28	3.61±1.22ab	0.67±0.67	4.33±2.40ab	2.28±1.71ab
H35	3.56±0.43ab	0.00±0.00	0.11±0.06	3.56±0.43ab	0.67±0.67	14.00±4.58ab	4.95±3.04ab
H36	3.83±0.54ab	0.33±0.25	0.22±0.22	4.17±0.42ab	0.33±0.33	1.67±0.88ab	0.71±0.38a
H37	4.72±0.39ab	0.17±0.17	0.33±0.33	4.89±0.24ab	0.67±0.67	11.00±6.11ab	4.65±3.88ab
H39	3.67±0.67ab	0.11±0.06	0.11±0.06	3.78±0.70ab	0.00±0.00	6.00±1.15ab	1.53±0.49ab
I1	3.00±0.67ab	0.06±0.06	0.00±0.00	3.06±0.73ab	0.00±0.00	1.67±0.88ab	0.31±0.21a
I2	2.33±0.92ab	0.22±0.15	0.17±0.17	2.56±1.04ab	0.00±0.00	2.00±0.58ab	0.34±0.17a
I4	4.06±0.56ab	0.11±0.06	0.22±0.15	4.17±0.51ab	1.33±0.88	5.00±2.65ab	2.48±1.73ab
I7	3.11±0.64ab	0.06±0.06	0.06±0.06	3.17±0.69ab	0.33±0.33	2.00±1.53ab	0.83±0.67a
I12	1.50±0.25ab	0.06±0.06	0.22±0.15	1.56±0.28ab	0.00±0.00	2.67±2.19ab	0.48±0.43a
I40	3.11±0.86ab	0.39±0.22	0.39±0.24	3.50±0.79ab	0.33±0.33	2.33±0.67ab	0.32±0.10a
I41	1.56±0.61ab	0.11±0.06	0.11±0.06	1.67±0.59ab	0.00±0.00	5.67±2.19ab	1.43±0.96ab
I42	1.67±0.44ab	0.11±0.11	0.06±0.06	1.78±0.43ab	1.67±0.88	6.33±1.86ab	2.92±1.28ab
I43	3.28±0.20ab	0.17±0.00	0.11±0.06	3.44±0.20ab	0.33±0.33	8.67±4.37ab	3.42±2.00ab
I44	3.72±0.45ab	0.33±0.19	0.17±0.17	4.06±0.53ab	1.33±1.33	29.67±10.97b	11.25±5.55b
I45	5.28±1.30b	0.00±0.00	0.00±0.00	5.28±1.30b	0.00±0.00	7.33±3.53ab	2.21±0.90ab
I46	4.22±0.78ab	0.00±0.00	0.17±0.17	4.22±0.78ab	1.00±1.00	8.00±0.58ab	2.44±0.73ab

F-Accession (A) [3]	1.869*	1.000	0.850	1.638*	1.170	2.141***	1.787*

1: Numbers are means ± standard errors (N = 3), lowercase letters indicate homogenous genotype groups based on Tukey LSD tests (P > 0.05). 2: Sample = 10 randomly selected plants. 3: F-values, accession DF = 31, Error Df = 64, * = $P \leq 0.05$ and *** = $P \leq 0.005$
